# Layered complexity, reorganisational ability and self-healing mechanisms of heteropolysaccharide solutions

**DOI:** 10.1038/s41598-024-64873-0

**Published:** 2024-06-17

**Authors:** Olena Ivashchenko

**Affiliations:** NanoBioMedical Centre, Adam Mickiewicz University in Poznań, Umultowska 85, 61614 Poznań, Poland

**Keywords:** Agarose, Gum arabic, Hyaluronic acid, Alginic acid, Self-healing, Reorganizational ability, Microstructure, Materials science, Soft materials, Self-assembly

## Abstract

Heteropolysaccharides are among the most widely distributed compounds in nature, acting as both tissue building blocks and as a source of nutrients. Their physicochemical and biological properties have been studied thoroughly; however, the microstructural properties of heteropolysaccharides are still poorly understood. This study aims to investigate the micro-structural peculiarities of agarose, gum arabic, hyaluronic and alginic acids by means of confocal laser scanning microscopy (CLSM) and cryogenic scanning electron microscopy (cryo-SEM). Herein, attention is paid to layered complexity of the microstructure differentiating surface, under surface, inner, and substrate interface layers. The scale and pattern of the polysaccharide’s microstructure depend on the concentration, changing from lamellae to cell-like porous structures. This work provides the insight into micro- and nanoscale mechanisms of self-healing and substrate-induced reorganisation. Thus, investigation of the self-healing mechanism revealed that this diffusion-based process starts from the fibres, turning into lamellae, following by cell-like structures with smaller dimensions. Investigation of the substrate-induced reorganisation ability showed that nano-to-micro (scale) porous substrate causes reorganisation in the interface layer of the studied heteropolysaccharides. This work contributes to understanding the structural peculiarities of heteropolysaccharides by looking at them through a supramolecular, micro-level prism.

## Introduction

In recent years, biological macromolecules, such as natural polysaccharides, have become a good choice for a range of biomedical and technological applications. Their wide availability as raw materials, good biocompatibility and water retention capacity make them an attractive candidate for such biomedical applications as cell culturing, wound dressings, biosensors, injectable scaffolds, drug carriers, etc.^1,2^ Moreover, they possess a mechanism of biodegradability and their degradation products are non-toxic to the biological objects and even can be absorbed by the human body as nutrients. These properties make them attractive materials for novel generation of consumables, such as food packing materials, membranes for water purification, etc.^3^ Polysaccharides containing more than one type of repeating monosaccharide in the polymeric chain (called heteropolysaccharides), are rich with polar functional groups such as amino, amido, hydroxyl, alkoxy, esters, etc. These groups can act as electron donors or adsorption sites. Due to this feature, heteropolysaccharides can bind with the metallic substrates forming protective layer, and act as an efficient corrosion inhibitors^4^. Thus, agar and alginates exhibit a strong anticorrosive activity for several metals and alloys, including aluminium, aluminium-copper alloys, and aluminium-air batteries^4–6^. The results indicate that combining of heteropolysaccharides with other functional compounds can provide a powerful synergistic effect and has a great potential.

For all above-mentioned applications, comprehensive investigation of the microstructural properties and behaviour are of great importance for the regulation of their chemical, physical, and biological performance. Some of the heteropolysaccharides, such as agar, alginates, hyaluronan may form gel—a soft matter with three-dimensional molecular network and high water capacity^7^. Many studies have been devoted to investigating the microstructure of heteropolysaccharides, their correlation with gelling, rheological and textural properties^7–13^. One of the first studies was published in 1972, where monochromatic optical rotation was used to monitor the structural changes in agar, galactomannan, and κ-carrageenan solutions^14^. The authors took into account that heteropolysaccharide average chain length of 30 to 60 disaccharide residues can undergo dimerization, and, thus, the gel structure results from an association of chain segments to form “junction zones” which are joined into a network. The optical rotation changes in polysaccharides were attributed to the formation and destruction of the chain associations—double- or triple-helix formations. Thus, according to this model inspired by the discovery of DNA structure in 1952, a gel is a network of chains organised in double helix formations^14^. This idea was further developed in 1979, when domain model of polymer gel structure was suggested^15^. In terms of this model, direct intermolecular association through double helices is confined to the formation of small independent domains involving a limited number of chains, and more long-range cross-linking occurs by association between helices in different domains^15^. Intermolecular association within these domains is through extended regions of ordered tertiary structure (double helices), while the elastic properties of gels also require the inter-domain quaternary structure (helix-helix aggregation). The other studies showed that a theoretical rheological model for agar gels, based on the double helices and higher order assemblies (suprafibers) correlates with experimental gelation curves obtained over a wide range of cooling rates (0.5–20 °C min^−1^) and agar concentration (1–3 wt%)^16,17^. The nanostructure of hydrogels from agar-based extracts has been also investigated using advanced small angle scattering techniques and rheology. The results of the measurements showing structural modification upon cooling were interpreted in terms of the association of agarose chains into double helices and bundles^18^.

Gellan gum, a linear anionic heteropolysaccharide, has been investigated in terms of gelation and effect on this process of the tetramethylammonium cation^11^. In this work, gelling and self-texturing properties of gellan gum were prescribed for the formation of the crystalline junction zones. Gellan gum molecules tend to form elongated molecular aggregates. Upon cooling, helix formation occurs and promotes end-to-end association into fibrils via double helix formation. These fibrils can thicken into fibres or bifurcate by a chain end-linking to the middle of a separate chain. Fibres, in turn, may arrange to form periodical crystalline junction zones. Such associations are responsible for the observed high viscosity and shear thinning behaviour of gellan gum samples. Herein, gel-promoting cations promote inter-fibril or intra-fibre crystallisation of gellan gum molecules, yielding a permanent network^11^. Another study investigated interactions between calcium ions and psyllium heteropolysaccharides^8^. The results suggested that there were strong interactions between Ca^2+^ and the heteropolysaccharide. These interactions contributed to the high viscosity, weak gelling property, and thermal stability of the psyllium heteropolysaccharide. The changes in the heteropolysaccharide properties were interpreted in terms of coordination bonds between Ca^2+^ and the polysaccharide molecules^8^.

Several studies investigated agarose fluid gels produced by gelation under shear, considering agarose as a set of microgel particles with "hairy" structures (dangling gel parts and chains on the particle surface)^9,10,19,20^. Herein, the biggest microgel particles are formed at the lowest agar concentration (0.5 wt%); with concentration increases, the size of microgel particles decreases^21^. The increase of the storage modulus with increasing agarose concentration was explained by increased particle interaction, especially the higher percolation between “hairy” structures forming helices and meshes that cross-link themselves by double helices^10^. According to this model, the agarose particles consist of cross-linked cores and looser surfaces (mesh, hair), and their size and shape with varying agarose concentration affect the bulk properties and friction behaviour. These studies have noticed the changes in agarose microstructure with concentration underlying an effect of microstructure on viscoelastic and flow behaviour^9,10,19,21^.

In recent decades, imaging techniques have been significantly improved, and a large volume of the results have been accumulated using different microscopic techniques^22–27^. Porous well-clustered network have been commonly detected for different polysaccharides, as well as fibrous and mesh-like microstructures^12,13,27–38^. However, the microstructure images of the same polysaccharide might differ significantly in scale and patterns from one study to another. Researchers focused most attention on the visualization of intrinsic microstructure but neglected layered complexity and influence of the interface interactions of the studied systems.

Reorganisational and self-healing abilities of natural polysaccharides are very attractive properties that can significantly prolong service life of polysaccharide-based material or coating, and are highly desirable for biomedical applications including drug delivery, wound healing, and tissue engineering^39,40^. Healing of defects is running without any external force in a way mimicking a small-area trauma healing in organisms. Self-healing processes can be driven by chemical (dynamic covalent bonds, reversible chemical reactions, dynamic imine bonds (dynamic Schiff base), disulfide bonds, reversible radical reaction, Diels–Alder reaction, etc.) or physical (dynamic non-covalent interactions, including hydrogen bonds, hydrophobic interaction, host–guest interaction and multiple intermolecular interactions) mechanisms^41–43^. Self-healing performance in hydrogels has been investigated by means of rheological analysis and optical microscopy in macro scale^39–41,44–47^. For quantitative estimation of the self-healing process, measurements of mechanical strength, elasticity and toughness have been applied^40,41,45,46^. However, micro- and nanoscale peculiarities of the self-healing process in the heteropolysaccharides have not been studied sufficiently.

This study aimed to investigate microstructural peculiarities of four natural heteropolysaccharides (agarose, gum arabic, hyaluronic and alginic acids), by means of two complementary microscopy techniques: cryogenic scanning electron microscopy (cryo-SEM) that produce the image of the frozen samples due to emission of the secondary electrons and confocal laser scanning microscopy (CLSM) that utilize fluorescence properties for sample visualization. Herein, special attention was paid to microstructure layers. Surface, subsurface, inner, expansive and transitional layers were differentiated. The reorganizational ability of the heteropolysaccharide solutions was investigated by examination of the interface at solution/porous substrate layer. Self-healing mechanism was investigated by examination of the incision site immediately after application and after 2 h of self-healing. The present study demonstrates concentration dependence of the microstructure scale and patterns, suggests the tubular domain model for the microstructure, and presents a scheme of the self-healing mechanism for the studied heteropolysaccharides.

## Results

**Figure 1:**
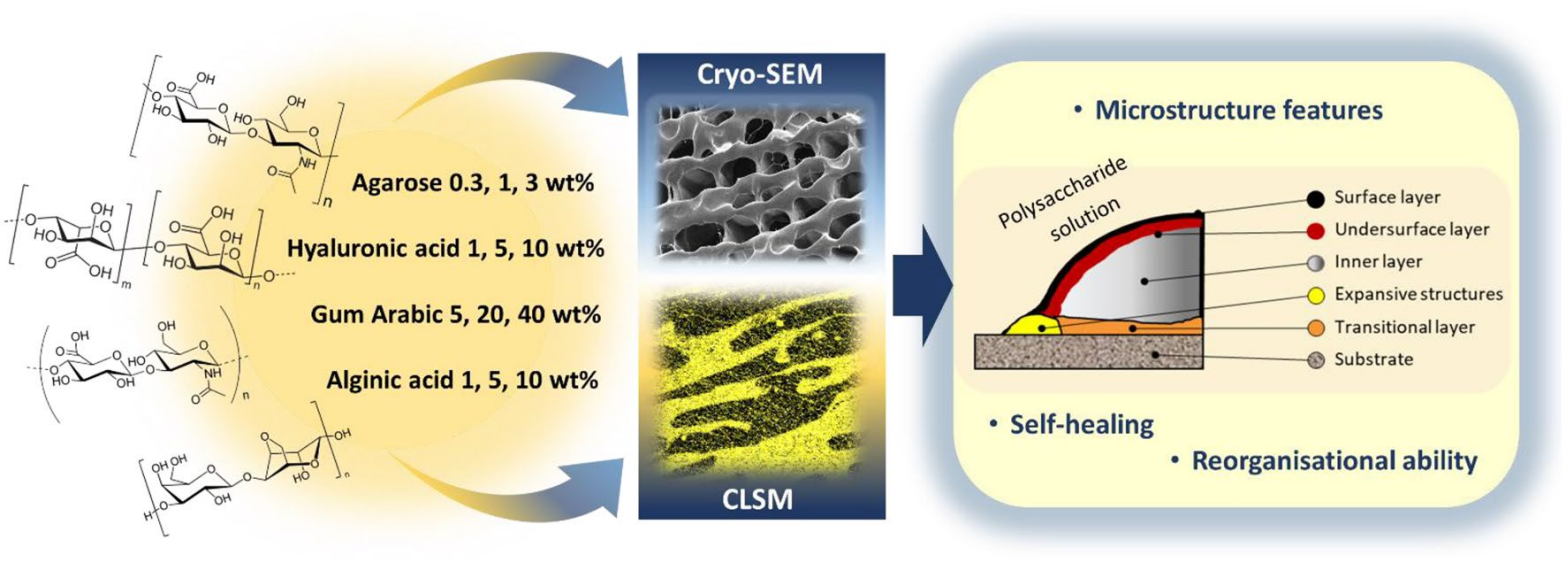
Scheme of the workflow—solutions of the four heteropolysaccharides (agarose, gum arabic, hyaluronic and alginic acids), at three concentrations were used for cryo-SEM and CLSM studies. Surface, under-surface, inner, transitional and expansive layers of their microstructure were investigated. Microstructure concentration dependence, self-healing and reorganizational abilities were also studied.

In the present study, agarose, gum arabic, hyaluronic and alginic acids were investigated from microstructural point of view by means of cryo-SEM and CLSM techniques at three concentrations each (workflow scheme in Fig. 1). Herein, the highest concentration was limited by heteropolysaccharide solubility, the lowest was eight or tenfold lower, and the other was an intermediate concentration. The heteropolysaccharides solutions were investigated considering that microstructures at the interface and surface are different from the bulk. The following microstructure layers were differentiated: surface (solution/air interface), subsurface (layer situated under surface, inner (bulk, approx. 10 µm far from interfaces), expansive structures (on the edges of solution/substrate interface) and transitional (at solution/substrate interface, near the substrate) layers.

Heteropolysaccharides used in this study are well-known and have international identification numbers for chemical substances (CAS, EC numbers). Their detailed physical and chemical characterisation can be found elsewhere^7,48–51^. FTIR study of the purchased heteropolysaccharides was performed in order to confirm their structural identity (Fig. S2a). Thus, intensive bands in the range of 1007–1036 cm^−1^ (C–O stretching, C–C stretching vibrations), 1366–1418 cm^−1^ (COO^−^ symmetric stretching, O‒H, C‒H bending), 1590–1634 cm^−1^ (COO^−^ antisymmetric stretching), 2857–2928 cm^−1^ (C‒H stretching), and wide broad band at 3229–3344 cm^−1^ (O‒H stretching) are characteristic bands for these heteropolysaccharides^52,53^. Morphological investigation of the powdered heteropolysaccharides performed by SEM (Fig. S2b) showed the irregular shape of the particles with a relatively smooth surface.

### Surface, subsurface, inner, and expansive structures

#### Agarose

**Figure 2:**
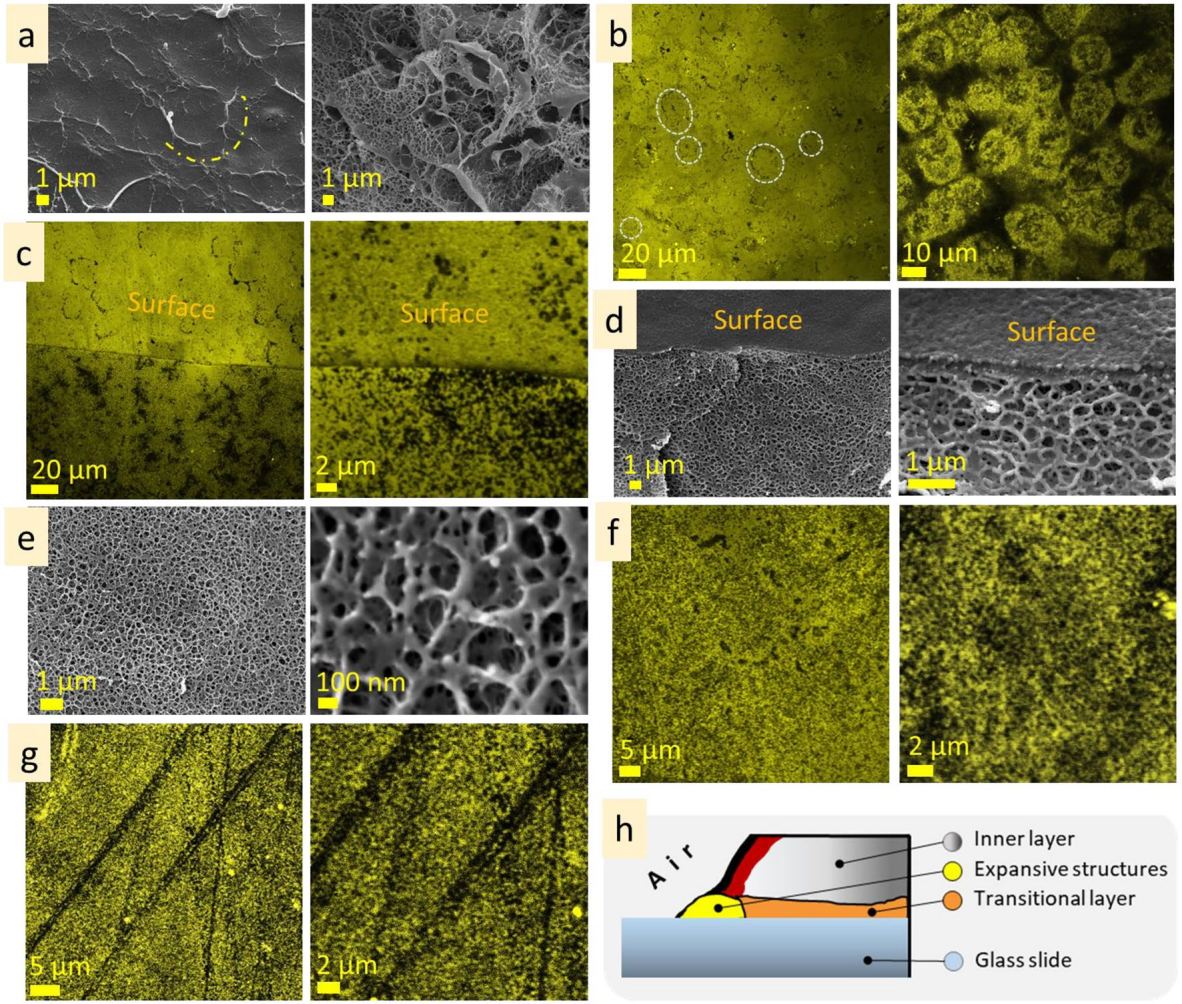
Microstructure of agarose (3 wt%): cryo-SEM and CLSM images of surface (**a** cryo-SEM; **b** CLSM), under surface layers (**c** CLSM; **d** cryo-SEM), inner (**e** cryo-SEM; **f** CLSM), expansive (**g** CLSM) layers. The scheme of the layers of the agarose solution microstructure (**h**).

The agarose (3 wt%) investigation by cryo-SEM revealed that its surface is rather continuous and dense, and some areas are covered with net-like structures. On the surface, there are contours of the roundish structures 5–10 µm in size (Fig. 2a, dot lines; Fig. S3a). These roundish structures may be mistaken for air bubbles, but they are not empty and contain a fine porous network inside, indicating them as a feature of microstructure. CLSM measurements also allowed us to observe similar roundish structures in some areas of the sample (Fig. 2b, dot lines; Fig.S2b). At CLSM measurements, it was noticed that the surface layer emits lower-intensity fluorescence than the inner microstructure. Investigation of the subsurface layer by cryo-SEM showed that it is thin, approx. 100 nm in depth (Fig. 2c; Fig. S3c). CLSM images show differences between surface and undersurface structure: the former is more continuous (Fig. 2d; Fig. S3d). Inner microstructure of agarose (3 wt%) solution is highly homogenous, spongy, with pore size 100–300 nm (Fig. 2e,f; Fig. S3e,f). During CLSM measurements, the linear structures near the border of the solution were observed (Fig. 2g). They are parallel and are approx. 1–2 µm width. These linear structures are referred as *expansive structures* further in the text (see scheme in Fig. 2h). The term *expansive structure* was chosen because these structures are predominantly near the border of a drop, at the air and specimen stub interfaces, where the solution tends to distribute (implement the expansion).

**Figure 3:**
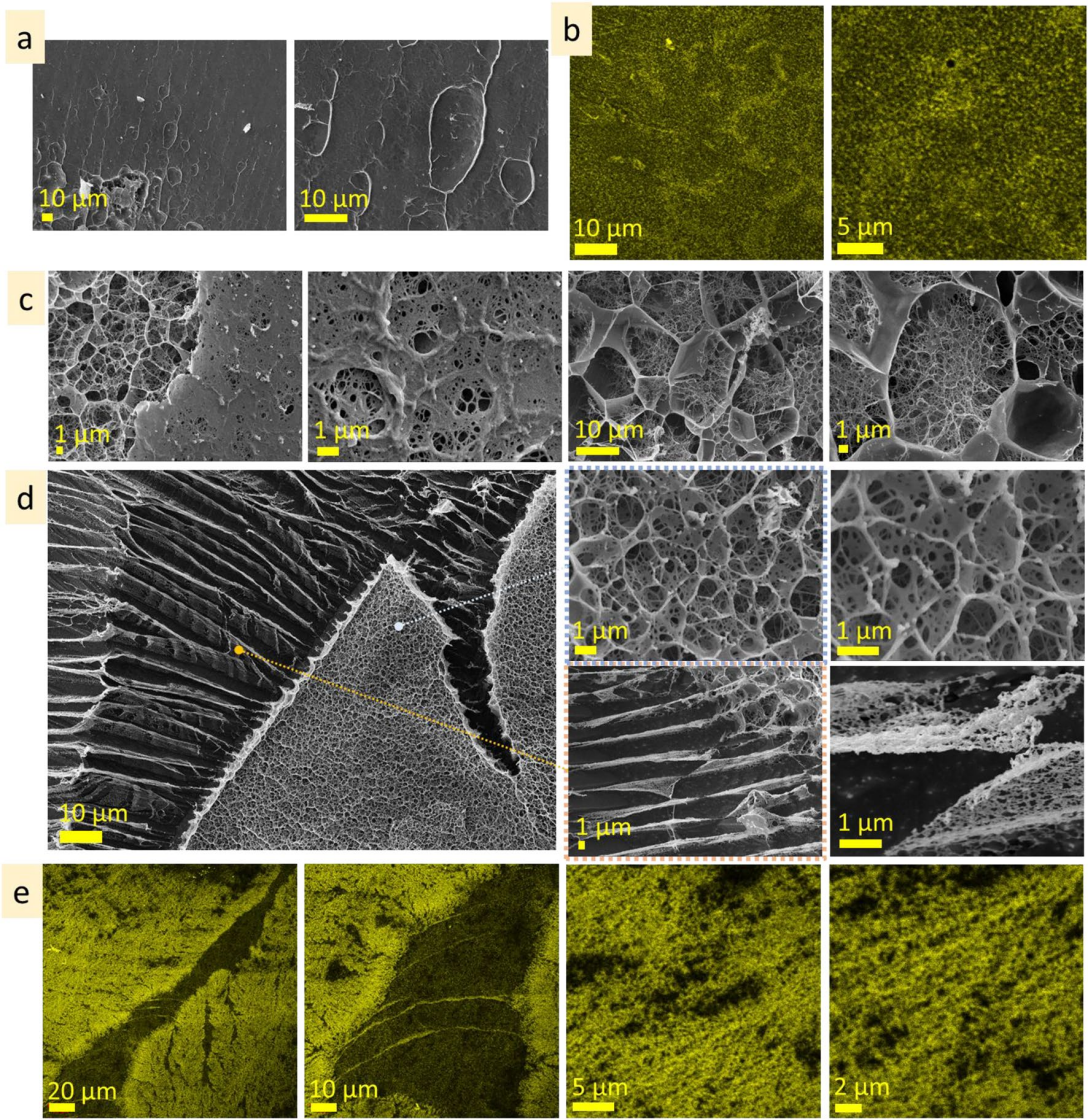
Microstructure of agarose (1 wt%): surface (**a** cryo-SEM; **b** CLSM), under surface (**c** cryo-SEM), inner (**d** cryo-SEM; **e** CLSM).

The surface of agarose (1 wt%) is continuous, homogenous and rather smooth, according to cryo-SEM and CLSM images (Fig. 3a,b; Fig. S4 a,b). Herein, the contours of the roundish shapes, similar to that observed for agarose (3 wt%), may also be noticed in cryo-SEM images (Fig. 3a). CLSM measurement revealed that the surface layer exhibits lower fluorescent intensity than the inner layer. The subsurface layer contains roundish structures, 5–30 µm in size, filled with fine porous network, ≤ 1 µm in scale (Fig. 3c). Inner layer of agarose (1 wt%) contains two types of microstructures—lamellae and porous (Fig. 3d,e; Fig. S4c,d). Herein, interlamellae distances are approx. 5–8 µm, whereas porous structure is finer (pore size approx. 1–2 µm).

**Figure 4:**
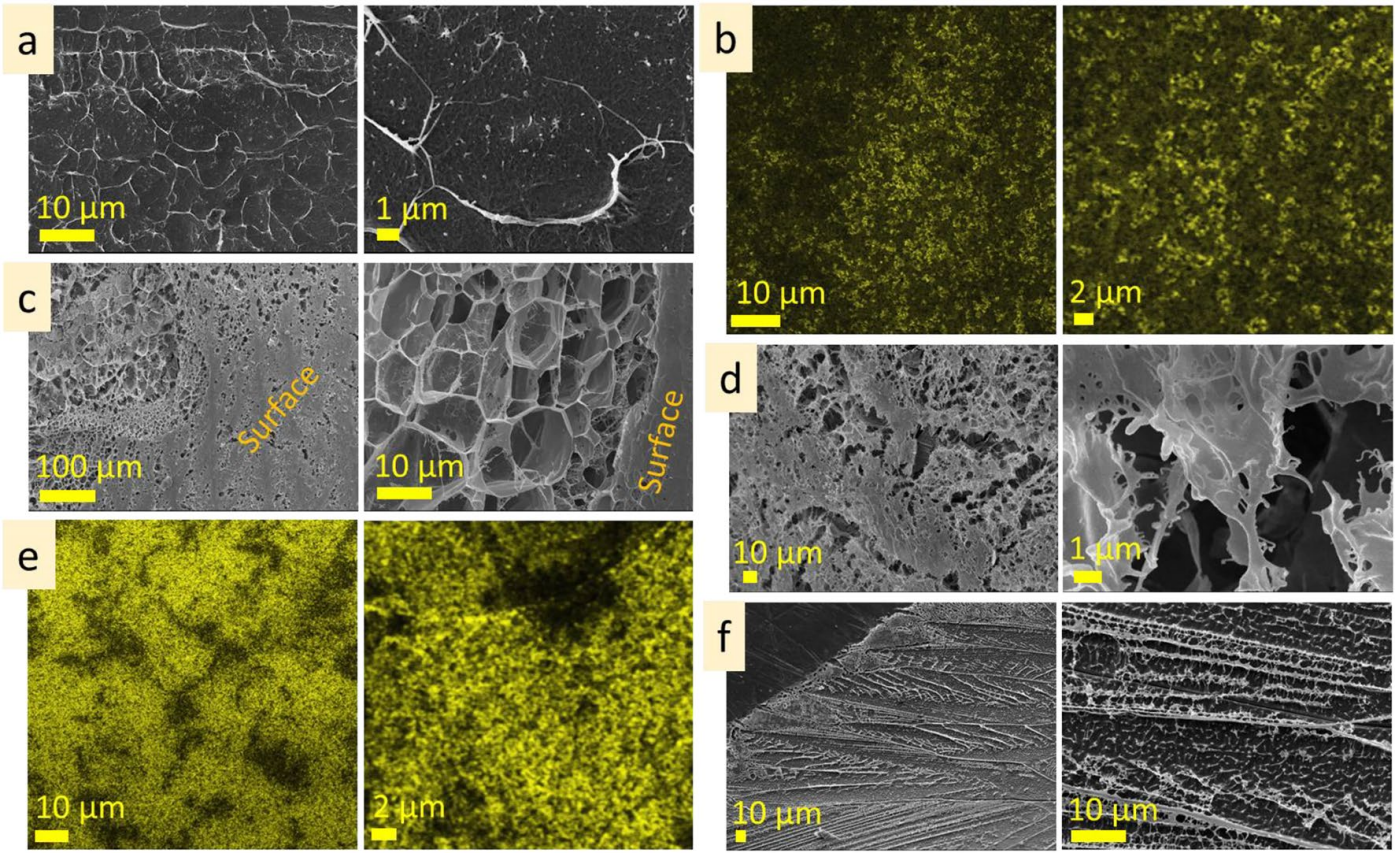
Microstructure of agarose (0.3 wt%): surface (**a** cryo-SEM; **b** CLSM), under surface (**c** cryo-SEM), inner (**d** cryo-SEM; **e** CLSM), expansive (**f** cryo-SEM) layers.

According to cryo-SEM, the surface layer of agarose (0.3 wt%) is continuous, homogenous, with arc patterns (Fig. 4a). CLSM images of the surface show a dotted pattern, and the intensity of fluorescent emission (in comparison with inner layer) is also significantly lower (Fig. 4b). Undersurface layer consists of irregular porous structure, with pore size up to 20 µm in diameter (Fig. 4c). Inner microstructure consists of irregular pores, approx. 5 µm in size, which distribution is not homogenous and has discontinuities (brakes) (Fig. 4d,e). Expansive structures at the solution border near the solution/substrate interface were also noticed during cryo-SEM measurements. They are linear structures that are arranged parallel or at an angle to each other (Fig. 4f, Fig. S5f). The width of these lines was ≤ 1 µm, and the intervals were from in the range of ten micrometres.

#### Alginic acid

**Figure 5:**
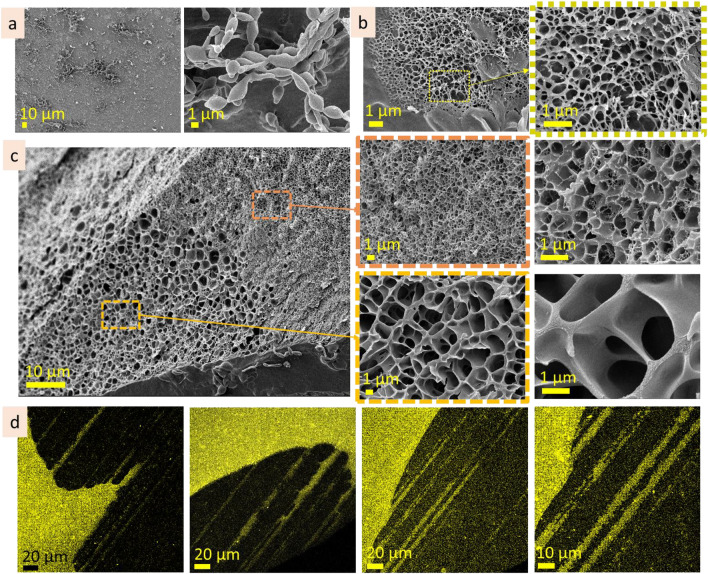
Microstructure of alginic acid (10 wt%): surface (**a** cryo-SEM), under surface (**b** cryo-SEM), inner (**c** cryo-SEM) and expansive (**d** CLSM) layers.

The surface layer of 10 wt% alginic acid is homogeneous, flat and has pieces of the chain-like structures on top (Fig. 5a, Fig. S6a). The subsurface layer predominantly consists of a porous structure with a pore size of 100–500 nm, but some subsurface areas have bigger pore size, approx. 3–10 µm (Fig. 5b, Fig. S6b). Inner microstructure is porous and forms domains hundreds of micrometres in size, with homogeneous pore size distribution (Fig. S6c). The dimensions of pores in these domains differ significantly: Fig. 5c demonstrates the coexistence of porous structures with different dimensions, smaller (pore size ≤ 500 nm) and bigger (pore size ≤ 2–10 µm). CLSM measurements showed homogenous fluorescent emittance at the glass slide interface, whereas at the solution border (at glass/solution/air interface) were observed expansive structures—linear structures that have parallel alighment (Fig. 5d). The width of these lines varied from 2 to 10 µm, and the intervals were from two to tens micrometres.

**Figure 6:**
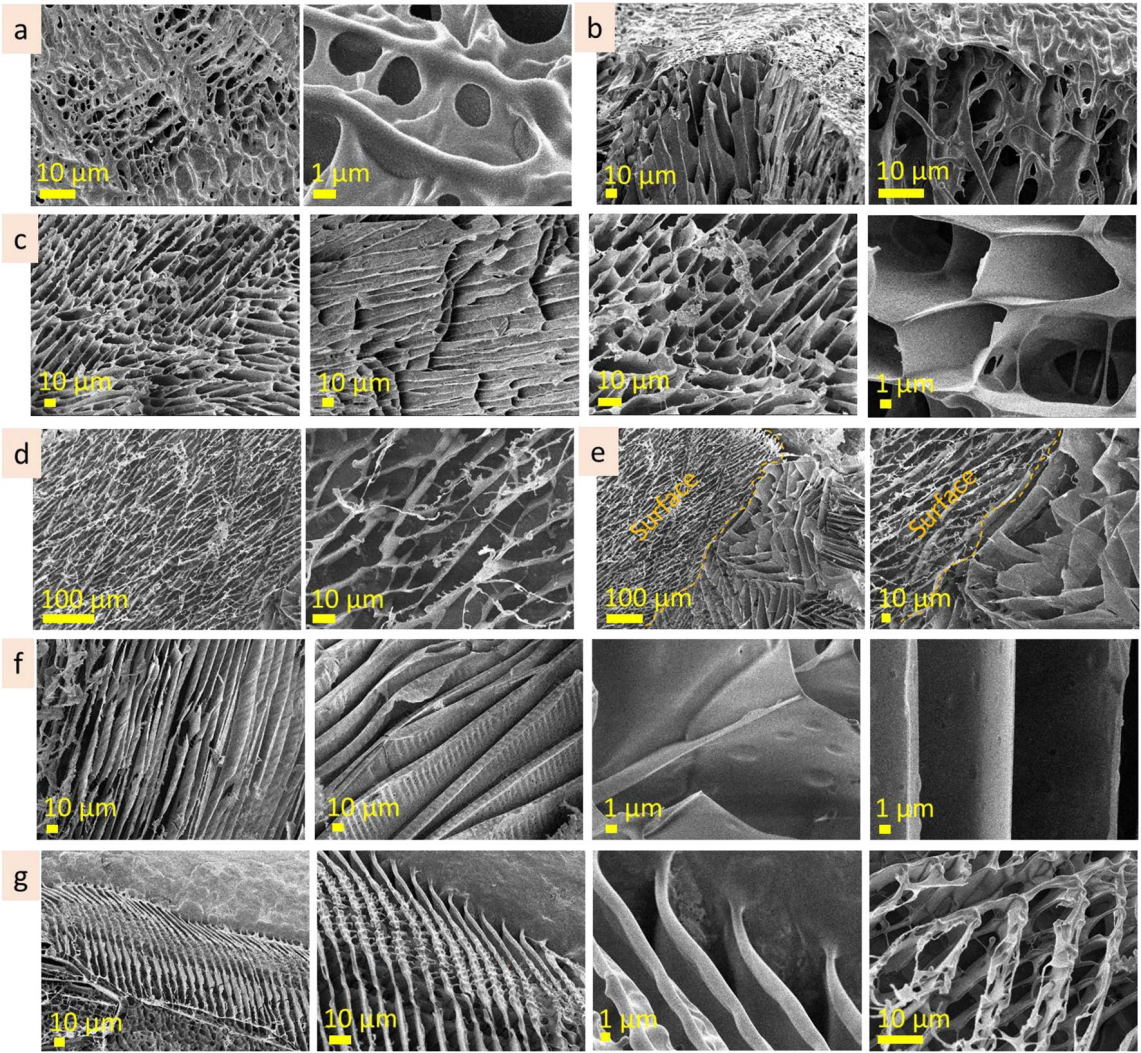
Microstructure of alginic acid (5 wt%): surface (**a**), under surface (**b**), inner (**c**) layers; alginic acid (1 wt%): surface (**d**), under surface (**e**), inner (**f**), expansive (**g**) layers (cryo-SEM).

The surface of the alginic acid 5 wt% is not flat and has relief pattern of approx. 5 µm in dimension (Fig. 6a, Fig. S7a). The subsurface layer, which provides a transition from fine surface structure to larger inner structure, is narrow (Fig. 6b, Fig. S7b). The inner microstructure is porous, with polygonal elongated pores ≤ 10 µm in width (Fig. 6c, Fig. S7c).

The surface of the alginic acid 1 wt% solution possesses a well-ordered complex microstructure with rhomboid patterns approx. 10 µm in scale (Fig. 6d, Fig. S8a). The subsurface layer provides the transition from surface to inner layers (Fig. 6e, Fig. S8b). The inner layer consists of lamellae structures with interlamellar distance approx. 10 µm (Fig. 6f, Fig. S8c). Figure 6g and Fig. S8d present cryo-SEM images of the expansive structures formed on the surface of the specimen stub. Here, expansive structures are three-dimensional parallel line structures with complex patterns that differ drastically from the inner layer.

#### Gum arabic

**Figure 7:**
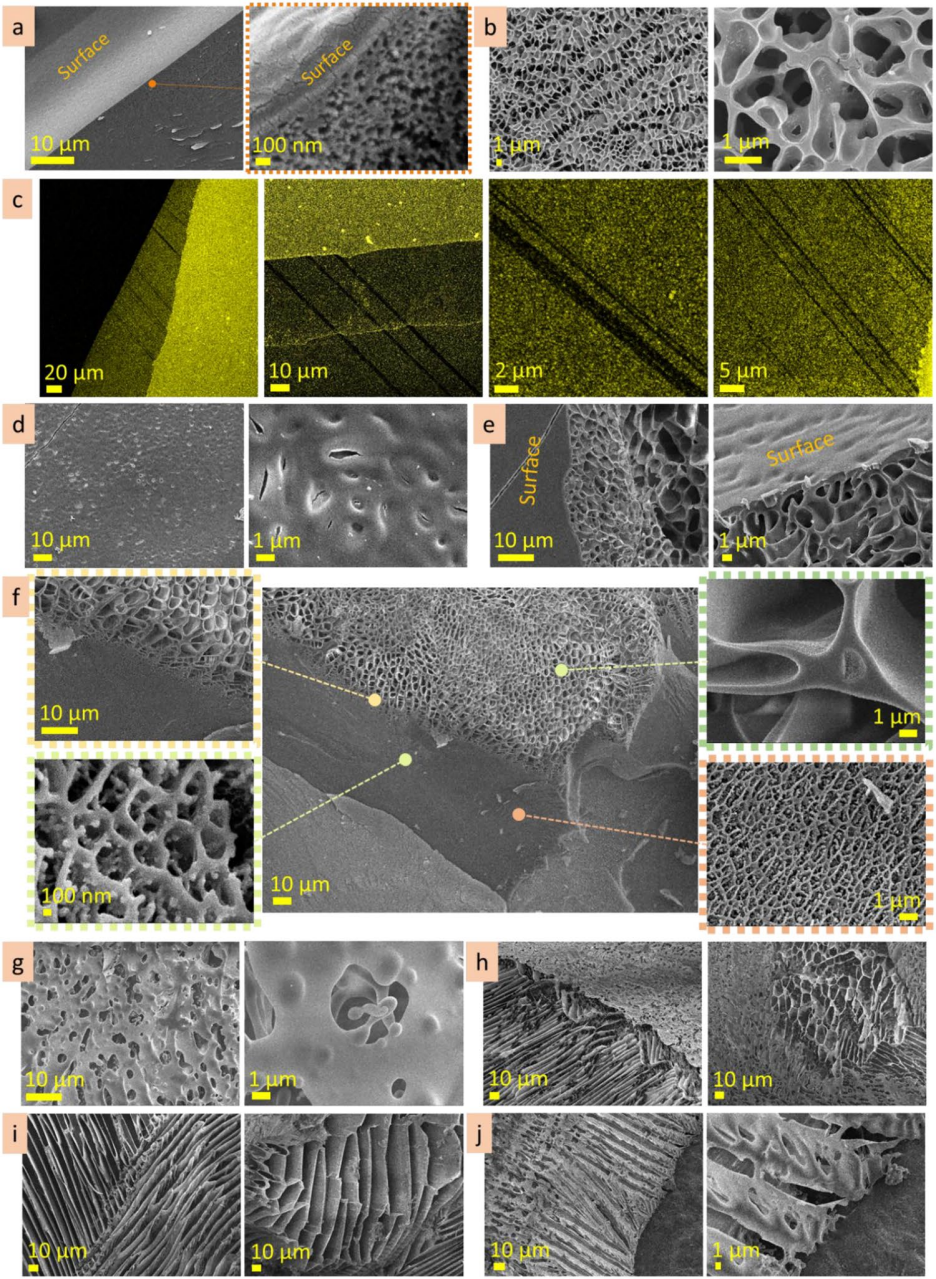
Microstructure of gum arabic (40 wt%): surface and subsurface (**a** cryo-SEM), inner (**b** cryo-SEM), expansive (**c** CLSM); gum arabic (20 wt%): surface (**d** cryo-SEM), subsurface (**e** cryo-SEM), inner (**f** cryo-SEM); gum arabic (5 wt%): surface (**g** cryo-SEM), subsurface (**h** cryo-SEM), inner (**i** cryo-SEM), expansive (**j** cryo-SEM) layers.

The surface of gum arabic (40 wt%) is continuous, flat, and homogenous (Fig. 7a). The depth of the subsurface layer is negligible. The porous microstructure of the inner layer is approx. 100 nm in scale (Fig. 7b), but it also has areas where the scale of pore size is bigger, from 1 to 3 µm (Fig. S9a). CLSM measurements revealed homogenous fluorescence emittance far from the solution edges, whereas parallel line structures (expansive structures) were noticed near the edges. The length of the line structures was in the range of hundred micrometres, and the width varied from 0.5 to 5 µm (Fig. 7c, Fig. S9b).

The surface of gum arabic (20 wt%) is smooth with dimpled patterns (Fig. 7d, Fig. S10a). The subsurface layer has a porous structure with pore size in the range of ≤ 3 µm near the surface, going in-depth, the pore size increases to approx. 6 µm in size (Fig. 7e, Fig. S10b). A feature of the inner layer is the coexistence of the areas (hundreds of micrometres in size) with different microstructure scales (Fig. 7f). Figure S10c shows the microstructure of the inner layer at the same magnification. As may be seen, the areas are similar in pattern, but the scale varied from 500 nm to approx. 10 µm.

The surface layer of gum arabic (5 wt%) is discontinuous and has holes (Fig. 7g, Fig. S11a). The subsurface layer provides a transition from porous to lamellae structure (Fig. 7h, Fig. S11b). Figure 7i and Fig. S11c present inner microstructure that consists of lamellae domains (a few hundred micrometers in size) oriented at an angle to one another. Interlamellar distance is in the range of 10 µm. Cryo-SEM images present expansive structures formed on a specimen stub (Fig. 7j, Fig. S11d). They have parallel linear structures that are several hundreds of micrometers long and approximately. 10–20 µm in width.

#### Hyaluronic acid

**Figure 8:**
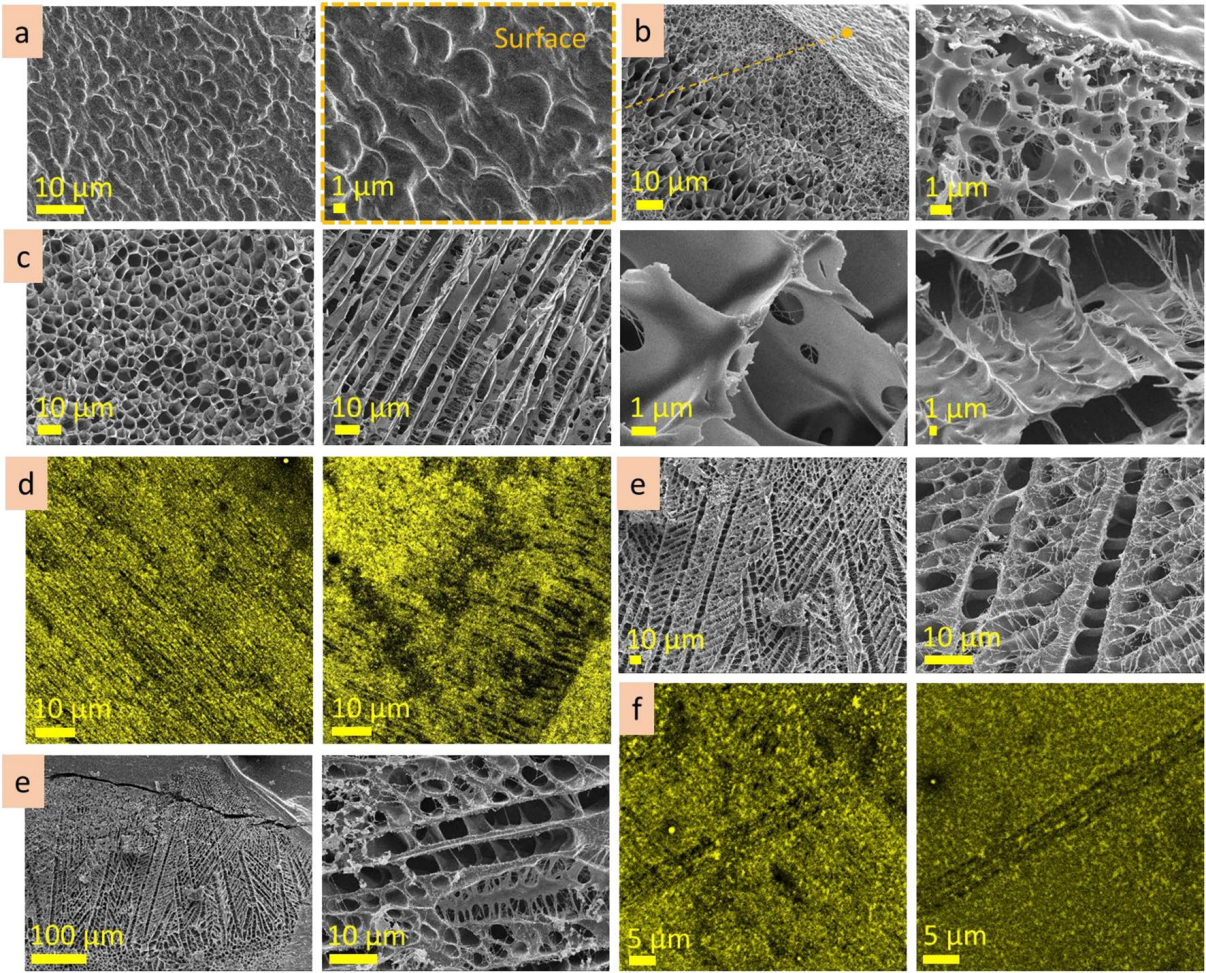
Microstructure of hyaluronic acid (10 wt%): surface (**a** cryo-SEM), under surface (**b** cryo-SEM), inner (**c** cryo-SEM; **d** CLSM), expansive structures (**e** cryo-SEM; **f** CLSM).

The surface of hyaluronic acid (10 wt%) is monolithic, with many hemispheres (3–8 µm in size) protruding above the surface (Fig. 8a). The subsurface layer extends to the depths of approx. 40 µm. It has fine porous structure (in the range of approx. 1 µm) closer to the surface and enlarges in the direction of the inner layer (Fig. 8b). The inner microstructure has a porous structure with polygonal pores that differ from one area to another being approx. 5–8 µm in size. Linear and lamellae microstructures with cross-bridges are also inherent for this solution (Fig. 8c,d; Fig. S12a, b). The interlamellar distance is approx. 5–8 µm. The observed porous, linear and lamellae structures are, presumably, tubular structures observed at different cross-section angles. Expansive structures formed on aluminum specimen stub and on a glass slide are shown in Fig. 8e,f and Fig. S12c.

**Figure 9:**
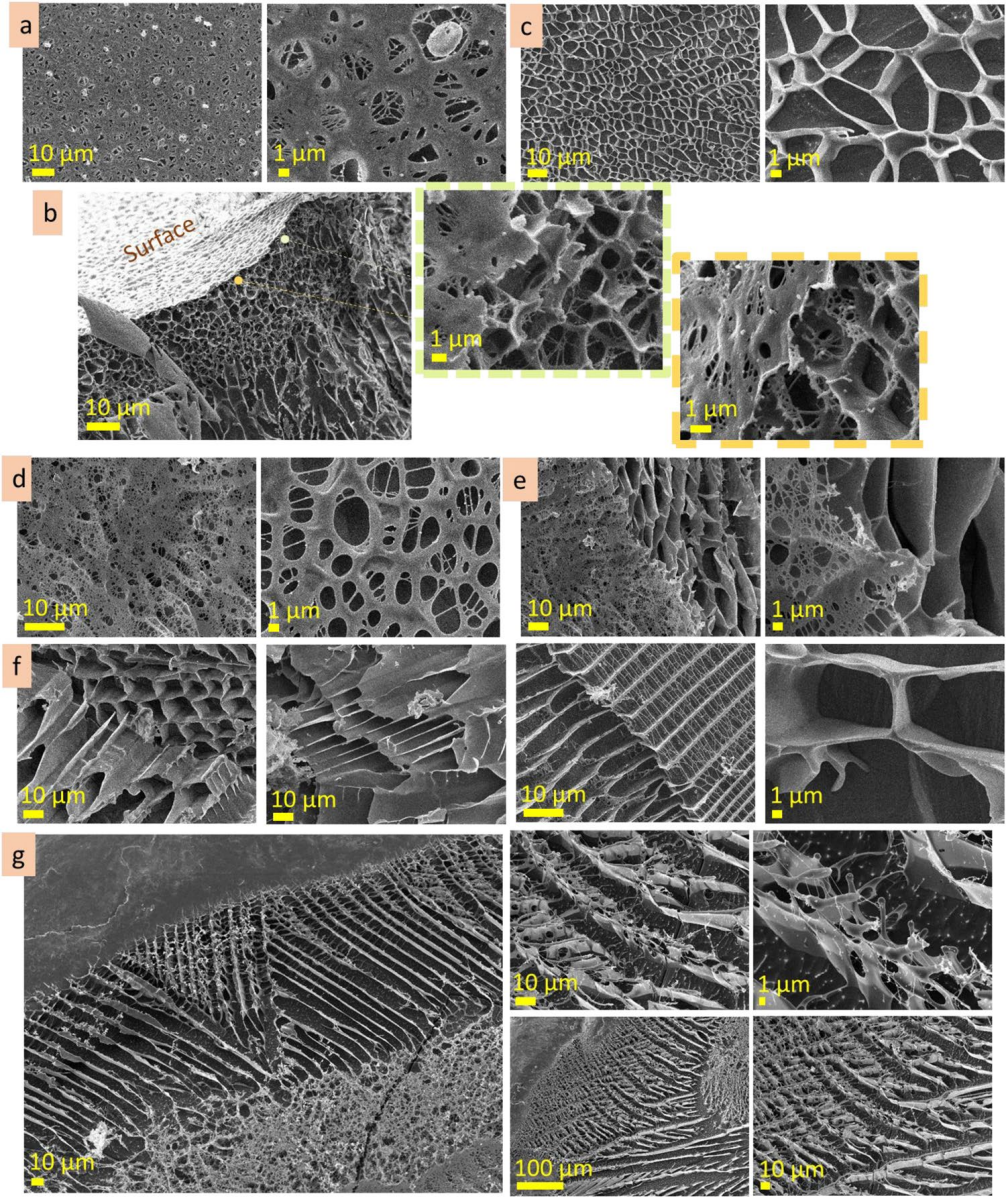
Microstructure of hyaluronic acid (5 wt%): surface (**a** cryo-SEM), under surface (**b** cryo-SEM), inner (**c** cryo-SEM) layers; hyaluronic acid (1 wt%): surface (**d** cryo-SEM), under surface (**e** cryo-SEM), inner (**f** cryo-SEM), expansive (**g** cryo-SEM) layers.

The surface of hyaluronic acid (5 wt%) is perforated by roundish holes (1–6 µm in diameter) with fibrous bridges (Fig. 9a, Fig. S13a). Undersurface layer is porous and has approx. 20 µm in depth. The pore size is smallest under the surface (approx. 1 µm), increasing gradually in depth towards the inner structure (Fig. 9b, Fig. S13b). Inner microstructure has pores with irregular size and shape, 6 ± 2 µm in size (Fig. 9c, Fig. S13c).

The surface of hyaluronic acid (1 wt%) is highly perforated and consists of a fibrous net (Fig. 9d, Fig. S14a). Subsurface layer has a smaller pore size just under the surface (approx. 5 µm) and is bigger in depth. Inner microstructure has linear cross-bridged areas and porous structures (Fig. 9e, Fig. S14b). The scale of these types of microstructures may differ—in one area interlamellar distance is 6 ± 1 µm, whereas in another 9 ± 2 µm; pore size was 5 ± 1 µm, but in another area it was 10 ± 2 µm. The examples of inner tubular structures are shown in Fig. 9f. The expansive structures at the edges of the solution are presented in Fig. 9g. Their width is in the range of 200–300 µm. The linear patterns of expansive structures differ from the inner layer. The periodicity of the linear patterns is approx. 10 µm.

### Self-healing

The self-healing mechanism was investigated using gelatinous heteropolysaccharide solutions that keep its shape when cut. For cryo-SEM measurements, a piece of gelatinous sample was fixed on a specimen stub and cut with a scalpel. The incision site was investigated at two intervals: a few seconds after cutting, and 2 or 3 h later, keeping the sample in a humid atmosphere at room temperature (21 °C). For CLSM measurements, a piece of jelly heteropolysaccharide solution was cut by a scalpel and put on a glass slide. Imaging the incision site was performed 2–3 h after cutting.

#### Hyaluronic acid 10 wt%

**Figure 10:**
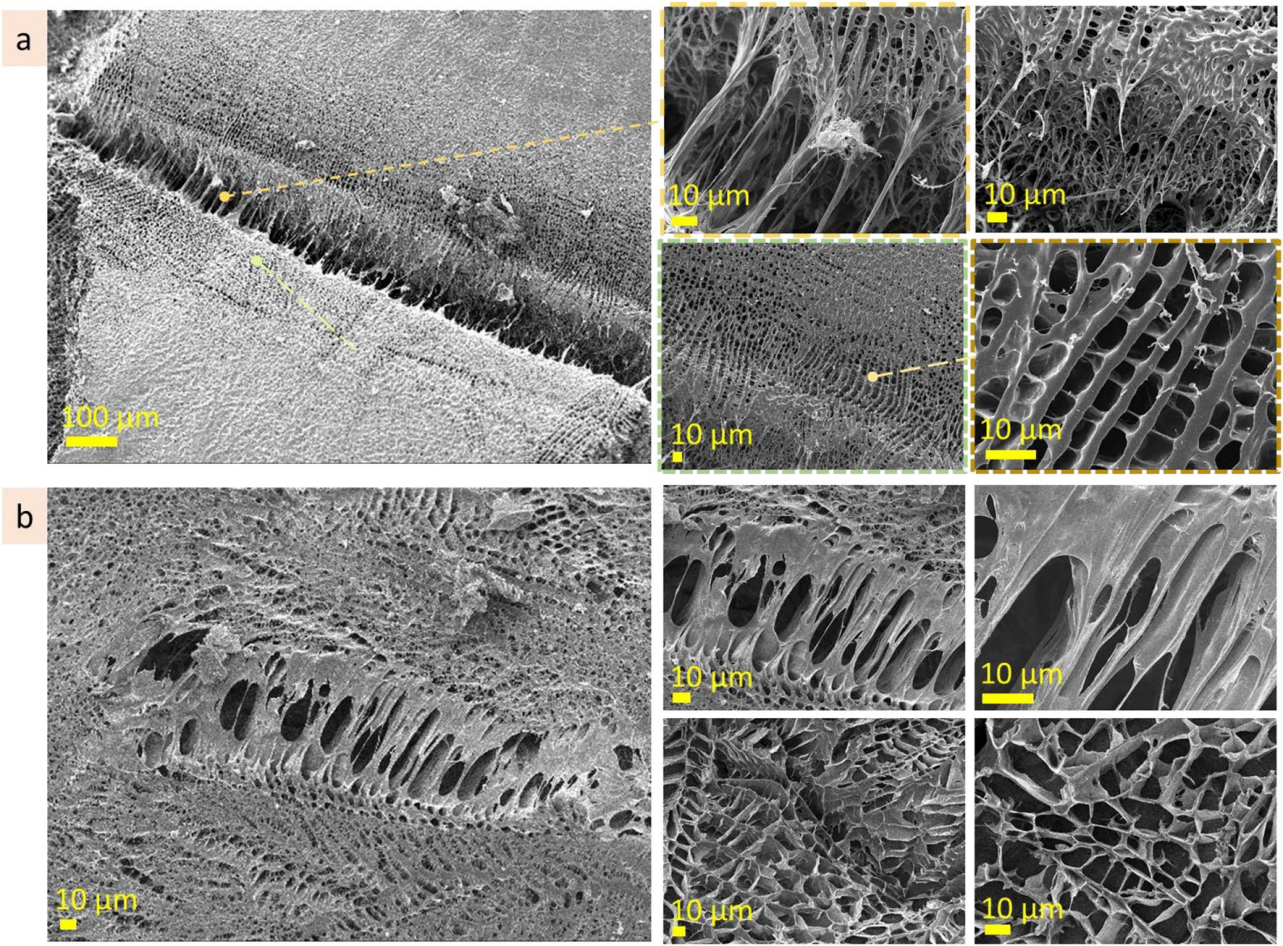
Self-healing: the incision site at hyaluronic acid (10 wt%) cut a few seconds before freezing (**a**), cut 2.5 h before freezing (**b**), cryo-SEM.

Being frozen seconds after cutting, the incision site at hyaluronic acid (10 wt%) solution has width in the range of 150 µm (Fig. 10a, Fig. S15). Inside the cut, besides broken porous structure there are fibers (2 ± 1 µm in diameter) connecting two sides of the section. Along the section, at both sides of it, there are regions (in the range of 50 µm in width) where the microstructure is rougher and has a bigger pore size. Supposedly, these regions may appear due to mechanical compression during cutting. These regions along the cut serve as a source for material needed for self-healing of the incision site. Being frozen 2 h after cutting, the incision site in hyaluronic acid (10 wt%) solution is discontinuous and untraceable in some parts (Fig. 10b). In some regions, the width of the incision site is 50–100 µm, in other the only traces with irregular pores can be noticed (Fig. 10b, bottom row). The fibers connecting the sides became thicker and served as a skeleton for plane lamellae structures forming at both sides of the section. In those regions where the cut is unspecified, porous structure with disturbed regularity appeared. 2.5 h after cutting, the pores in those regions had irregular shape and size in the range 14 ± 4 µm.

#### Agarose 3 wt%

**Figure 11:**
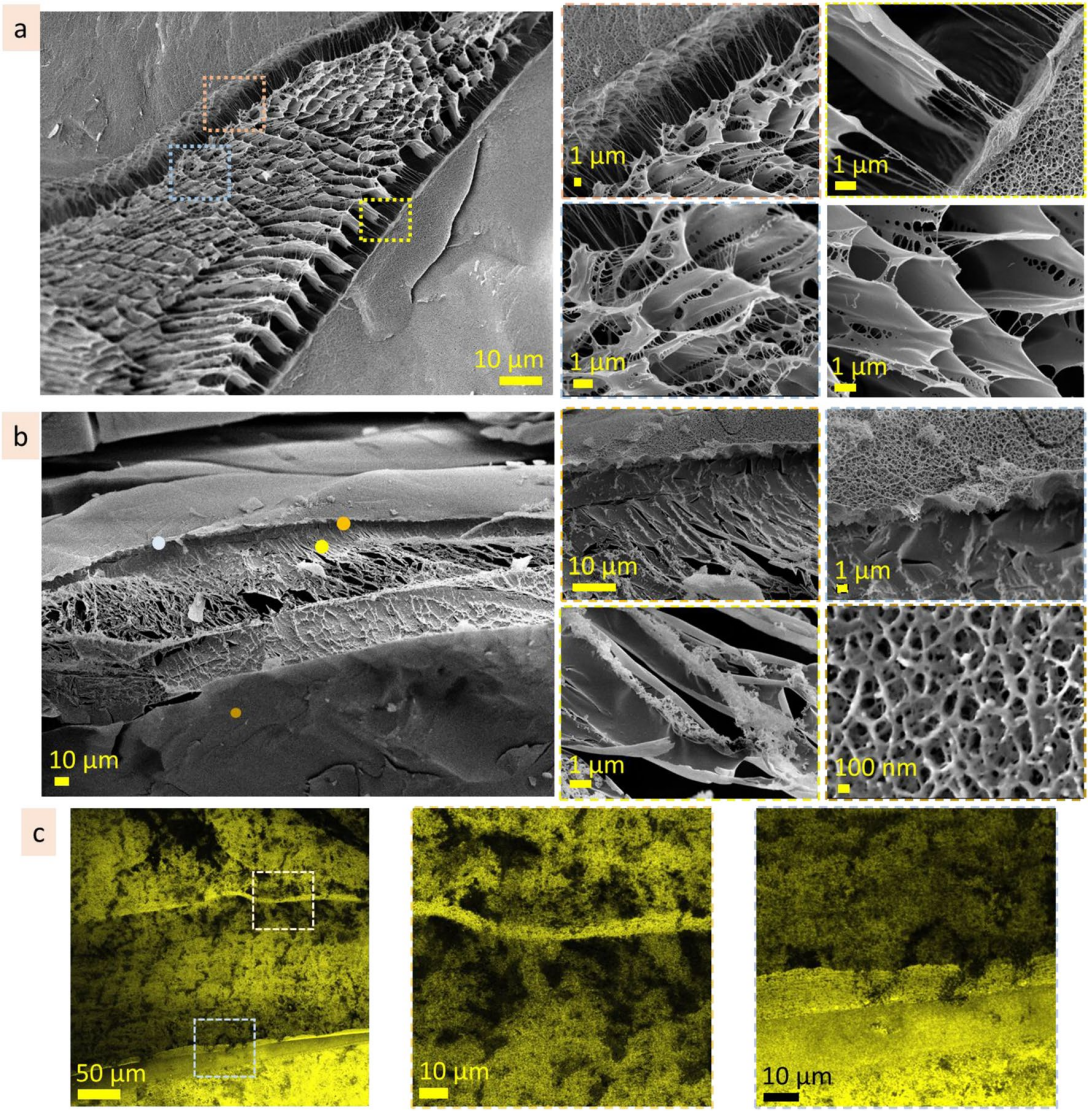
Self-healing: the incision site at agarose (3 wt%) a few seconds after cutting (**a** cryo-SEM), 2.5 h after cutting (**b** cryo-SEM), CLSM images (**c**).

Seconds after cutting, the incision site in agarose 3 wt% solution is in the range of 140 µm in width, being wider or narrower in some areas (Fig. 11a, Fig. S16a). Some parts of the incision site are filled with transverse linear structures. These structures are few microns thick and cross-bridged by nanoscale fibers. The other parts of the incision site are connected by nanoscale transverse fibers or filled with tubular microstructure which size is 6 ± 1 µm and is significantly bigger than pore size in the neighboring non-damaged microstructure. 2.5 h after cutting the incision site in agarose 3 wt% is steel noticeable and has similar width (Fig. 11b, Fig. S16b). It is filled by microstructure with significantly bigger dimension than neighboring areas. In the cut, the microstructure is irregular and has features of both linear and porous structures. From the one side along the cut, there is a strip region, approx. 40 µm in width, which microstructure differs from undamaged neighboring areas. This border region serves as a source of material for self-healing. Diffusion of agarose from this area into the incision site causes changes in microstructure. CLSM imaging of the incision site of agarose (3 wt%) allowed to determine differences in microstructure of the incision site, its borders, and a strip region along the border of the incision site (Fig. 11c, Fig. S16c).

#### Agarose 1 wt%

Seconds after cutting, the incision site of agarose 1 wt% is filled with fibrous linear structures with parallel alignment connecting two borders of the incision site. They have approx. 0.5 µm thick and are located with an interval of 6 ± 2 µm (Fig. S17a). CLSM imaging of the incision site of agarose 1 wt% revealed differences in microstructures of inner, incision site, borders areas and a strip region along the border (Fig. S17b).

Based on the above observations, the self-healing of agarose and hyaluronic acid gels started from nanoscale fibres, forming two sides of the cut. The agarose molecules diffuse through these fibers into the incision site expanding and developing a substitutional microstructure. The development of substitutional microstructure, which occurred 2–3 h after cutting, started from the formation of nanoscale fibres followed by the appearance of lamellae planes, then lamellae planes with cross-bridges, and resulted in the formation of the irregular porous structure that substitutes the section. The substituting microstructure is usually bigger in scale and has irregularities in shape and size. During the next hours, the microstructure will probably become homogeneous because of the continuous diffusion processes. The diffusion of molecules into the incision site goes from stripe region (approx. 50 µm in width) along one side of the cut edge. This strip region providing resources for substitutional structure is clearly defined and not blurred. In time, the microstructure in this region became bigger in scale due to the local decrease of concentration. Interestingly, that only one side of the cut becomes a dominant donor for microstructure recovery.

### Reorganisational ability

The reorganisational ability of the heteropolysaccharides was investigated using porous substrate-acid-treated specimen stub, on which a drop of the heteropolysaccharide solution was exposed (2 h 40 min, 21 °C, increased humidity) before cryo-SEM measurements. Control experiments were performed using non-acid-treated specimen stub (as received from the manufacturer) following the same protocol of the experiment. During the cryo-SEM measurements, attention was paid to the stub surface/solution interface. For the convenience of the readers, the microscopic images were coloured: substrates in blue, heteropolysaccharide solutions in beige. The microscopic images of the initial and acid-treated specimen stubs are shown in Fig. S18. As seen, the surface of the initial stub (used for control experiments) has some roughness in nano- and micro-scales—the traces of surface polishing. Acid-treated surface has significantly bigger surface irregularities and indentations in the surface, which are hundreds of micrometres in size. This surface is porous from nano to micro scales. Because of the big difference in roughness, the initial stub will be referred to as “smooth” and acid-treated—as “porous” substrates.

#### Agarose (0.3 wt%)

**Figure 12:**
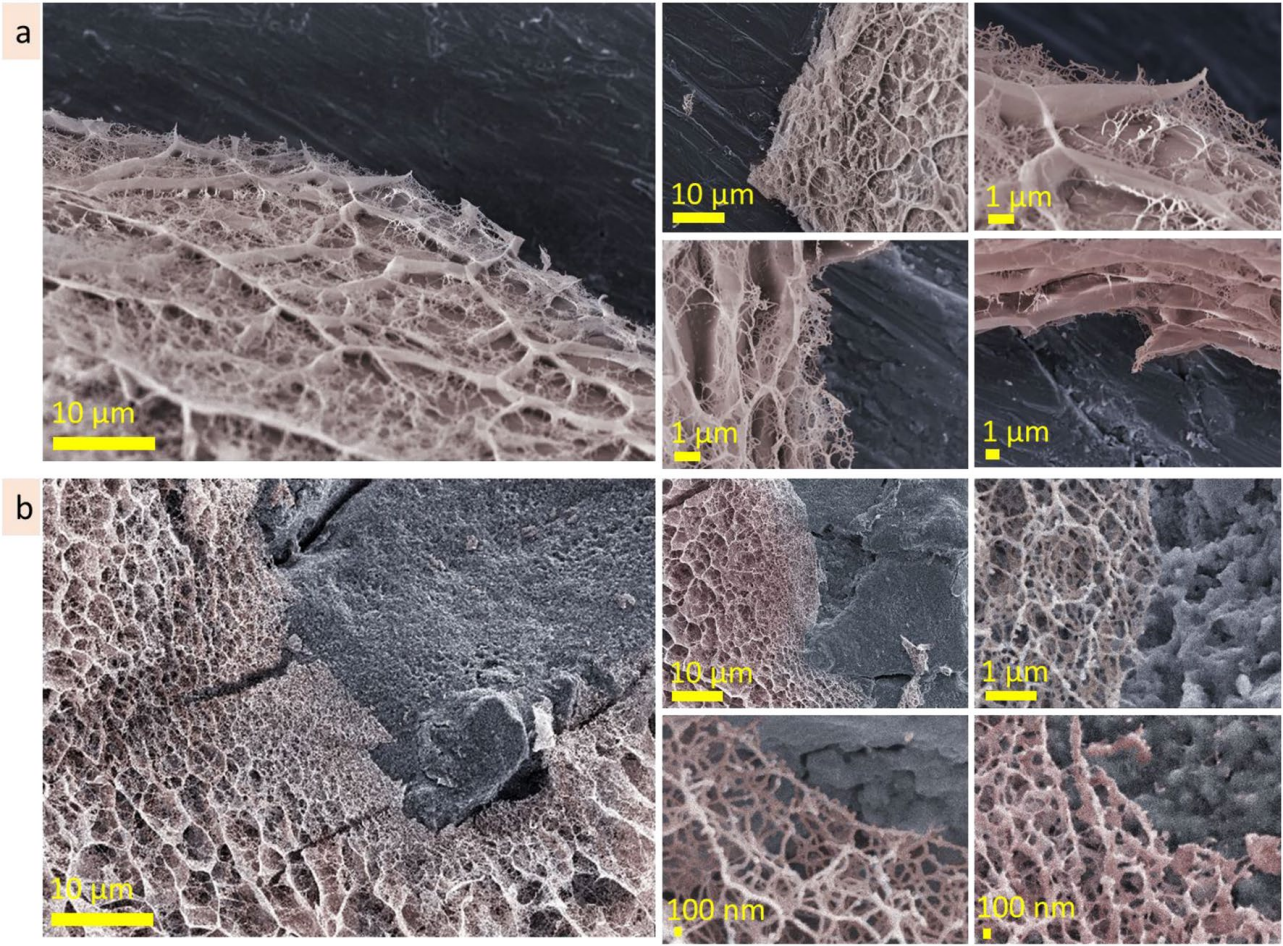
Reorganisational ability: cryo-SEM coloured images of agarose (0.3 wt%) microstructures after exposition on smooth (**a**) and porous (**b**) substrates. Blue colour corresponds to substrate, beige—to heteropolysaccharide microstructures.

The microstructure of agarose (0.3 wt%) after exposition on the smooth surface (Fig. 12a, Fig. S19a) has a network of the bigger elements (approx. 10 µm in size) filled with fine net structure with cell size in the range of 500 nm. The scale of microstructure does not change significantly going from substrate interface to the above inner microstructure. When agarose (0.3 wt%) was exposed on porous substrate, a gradient in the microstructure scale appeared (Fig. 12b, Fig. S19b) going from the interface to the bulk. Thus, the size of the microstructure elements is smaller near the porous surface and increases with distance from it. At the interface, the microstructure of agarose (0.3 wt%) is a network with cell size in the range of 200–500 nm and looks proportionally to the surface topology. Away from the porous surface, the scale of the microstructure increases to approx. 6–8 µm. These results show that agarose can adjust its microstructure to the surface topology.

#### Alginic acid (5 wt%)

**Figure 13:**
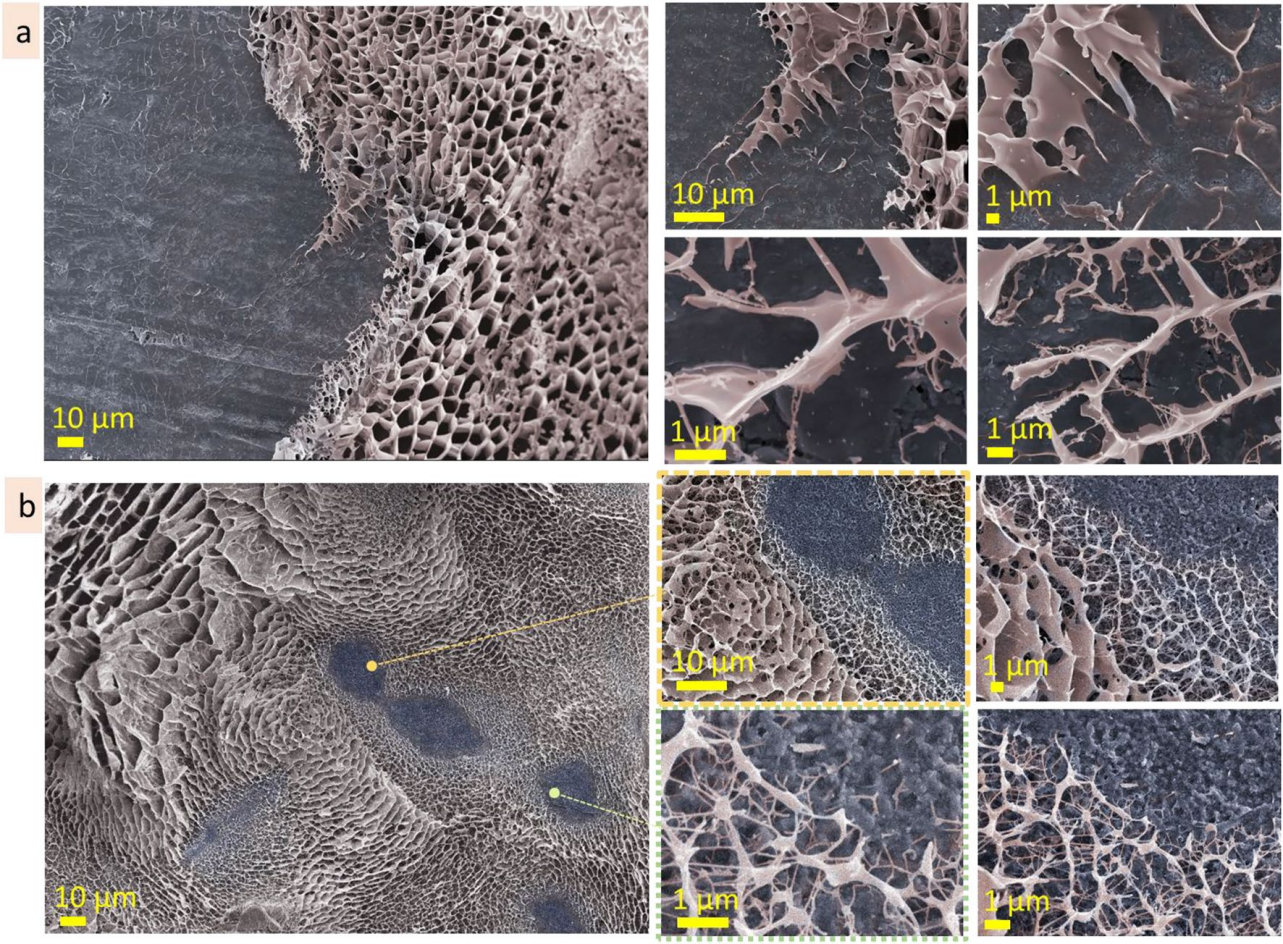
Reorganisational ability: cryo-SEM coloured images of alginic acid (5 wt%) microstructures after exposition on smooth (**a**) and porous (**b**) substrates. Blue colour corresponds to substrate, beige—to heteropolysaccharide microstructures.

Being exposed on the smooth substrate, the interface microstructure of the alginic acid has mainly a scale in the range of 5 ± 2 µm (Fig. 13a). There are also some areas with smaller scale, in the range of 0.6–1 µm (Fig. S20a). A gradient in microstructure scale, from smaller at the interface to bigger in bulk, was noticed. When exposed on porous substrate, the gradient in the microstructure scale become more pronounce (Fig. 13b). At the porous interface, the scale is ≤ 1 µm, whereas going away from the interface the scale increases, and at the distance approx. 100 µm, the size of pores becomes 10 ± 2 µm. Two images in Fig. S20b, on the right, show how the alginic acid cover a hole in the substrate. These results indicate that the microstructure of the interface layer depends on the substrate topology. Roughness of the substrate forces the appearance of the transitional gradient layer. Through this layer, the alginic acid microstructure adopts scale and patterns to the substrate topology and transits into the regular one. The bigger roughness of the substrate, the bigger gradient in the transitional layer.

#### Gum arabic (20 wt%)

**Figure 14:**
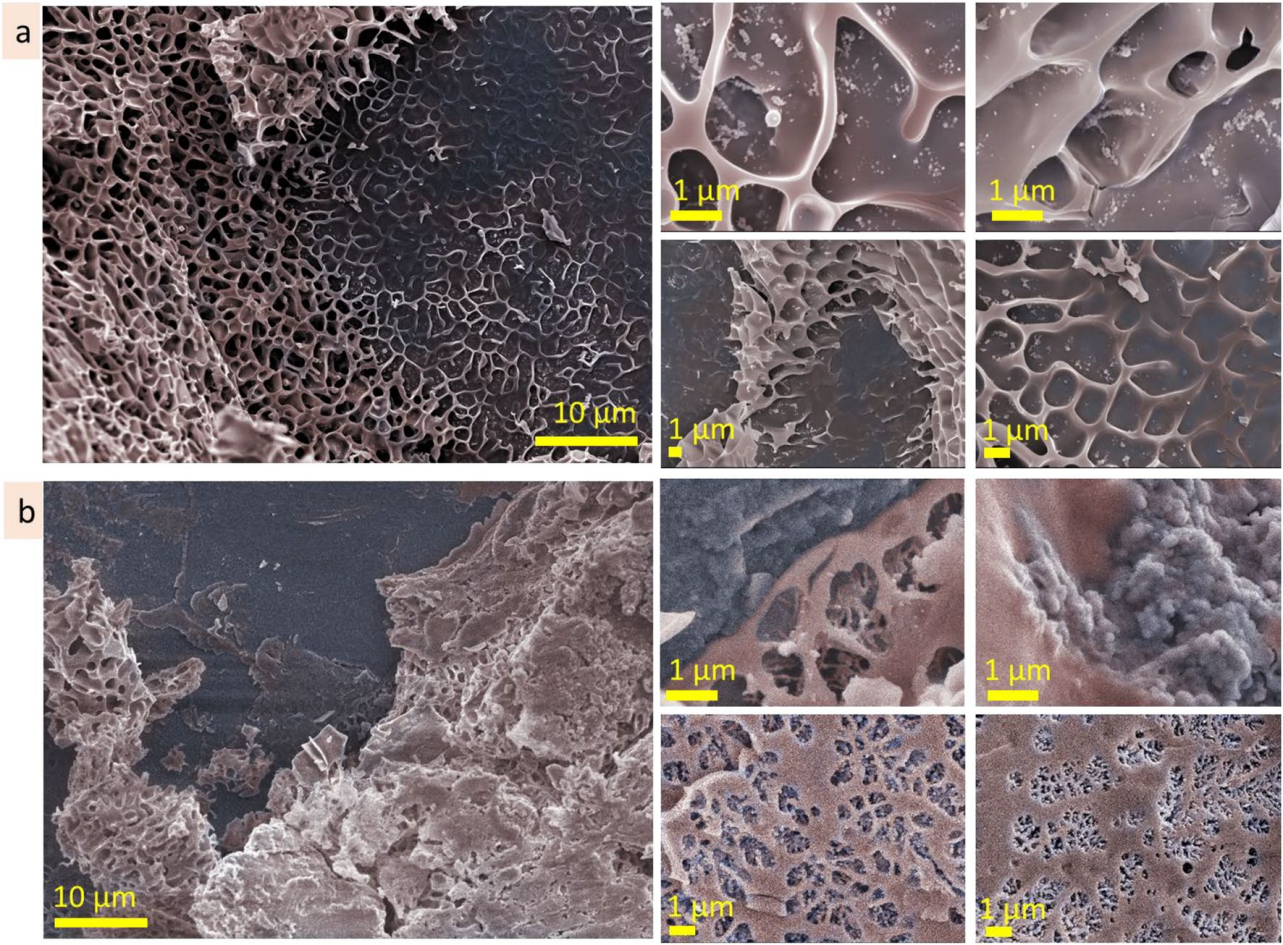
Reorganisational ability: cryo-SEM images of gum arabic (20 wt%) microstructures after exposition on smooth (**a**) and porous (**b**) substrates. Blue colour corresponds to substrate, beige—to heteropolysaccharide microstructures.

On the smooth surface, gum arabic (20 wt%) forms porous structure with pore size in the range of 3 ± 1.5 µm (Fig. 14a, Fig. S21a). Being exposed on the porous substrate, it forms finer porous structure with pore size 1 ± 0.5 µm. Some areas, however, have bigger pore size that may be prescribed to the surface inhomogeneity in microscale. The microstructure of the interfacial layer is similar in scale to the roughness of the substrate topology. A transitional interface layer of the gum arabic solution smooths the roughness of the substrate (Fig. 14b, Fig. S21b).

#### Hyaluronic acid (1 wt%)

**Figure 15:**
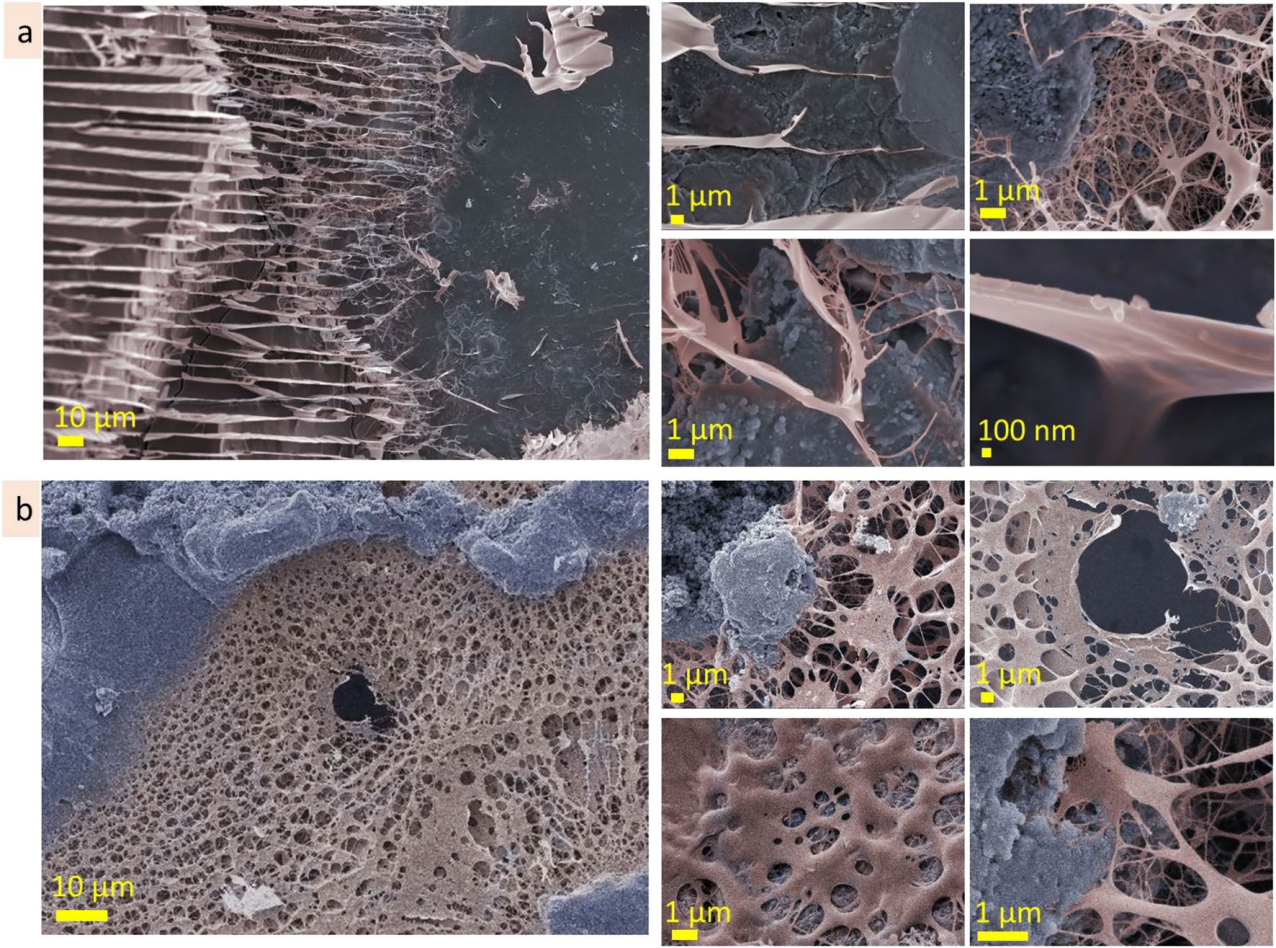
Reorganisational ability: cryo-SEM images of hyaluronic acid (1 wt%) microstructures after exposition on smooth (**a**) and porous (**b**) substrates. Blue colour corresponds to substrate, beige—to heteropolysaccharide microstructures.

Being exposed on the smooth substrate, hyaluronic acid (1 wt%) form predominantly lamellae structures with interlamellar distance approx. 7 ± 2 µm (Fig. 15a, Fig. S22a). Some areas, probably due to the local defects of the surface, have smaller scale net-like structures (≤ 1 µm in scale). Exposition on the porous substrate caused the formation of the transitional layer, approx. 100 µm in depth. In this layer, pore size is changed from ≤ 1 µm to 7 ± 2 µm (Fig. 15b, Fig. S22b). These results indicate that surface inhomogeneities may force the microstructure of the hyaluronic acid.

To summarise, the microstructure of the heteropolysaccharides responds to the roughness of the substrate topology forming transitional layer. The width, pattern and scale of the transitional layer depend on the size of topological elements (pores, indentations, holes, etc.) of the surface. The bigger roughness of the substrate, the wider transitional layer and bigger gradient in scale.

## Discussion

In this study, attention was paid to the differences between the layers of microstructure of the heteropolysaccharide solutions. The applied techniques, cryo-SEM and CLSM, have different possibilities for the observation of microstructure layers. In particular, during CLSM measurements, a few micrometres layer above the glass slide is investigated. This layer is in fact an interface layer that includes solution/glass and air/solution/glass interfaces. The influence of the interface interactions on the microstructure cannot be neglected. For any heteropolysaccharide solution, in time, at the solution/glass interface, a layer with a transitional microstructure (transitional layer) that depends on physicochemical interactions, surface properties and chemical nature of a glass slide. At the air/solution/glass interfaces (at the edges) appeared a characteristic linear microstructure (referred as “expansive layer”), which is responsible for spreading of the solution on the substrate. Thus, it should be taken into account that during CLSM measurements, we observe mainly transitional and expansive layers of heteropolysaccharide solutions (scheme in Fig. S23). Cryo-SEM technique provides wider possibilities for investigation of different microstructure layers because of the possibility of fracturing the specimen before observation. Depending on the aim, a researcher can fracture the specimen in a way suitable for observing the surface, subsurface, inner layers or transitional layer, which is situated above the substrate. The combination of these two techniques, CLSM and cryo-SEM, benefited in understanding the heteropolysaccharide solutions microstructures.

**Figure 16:**
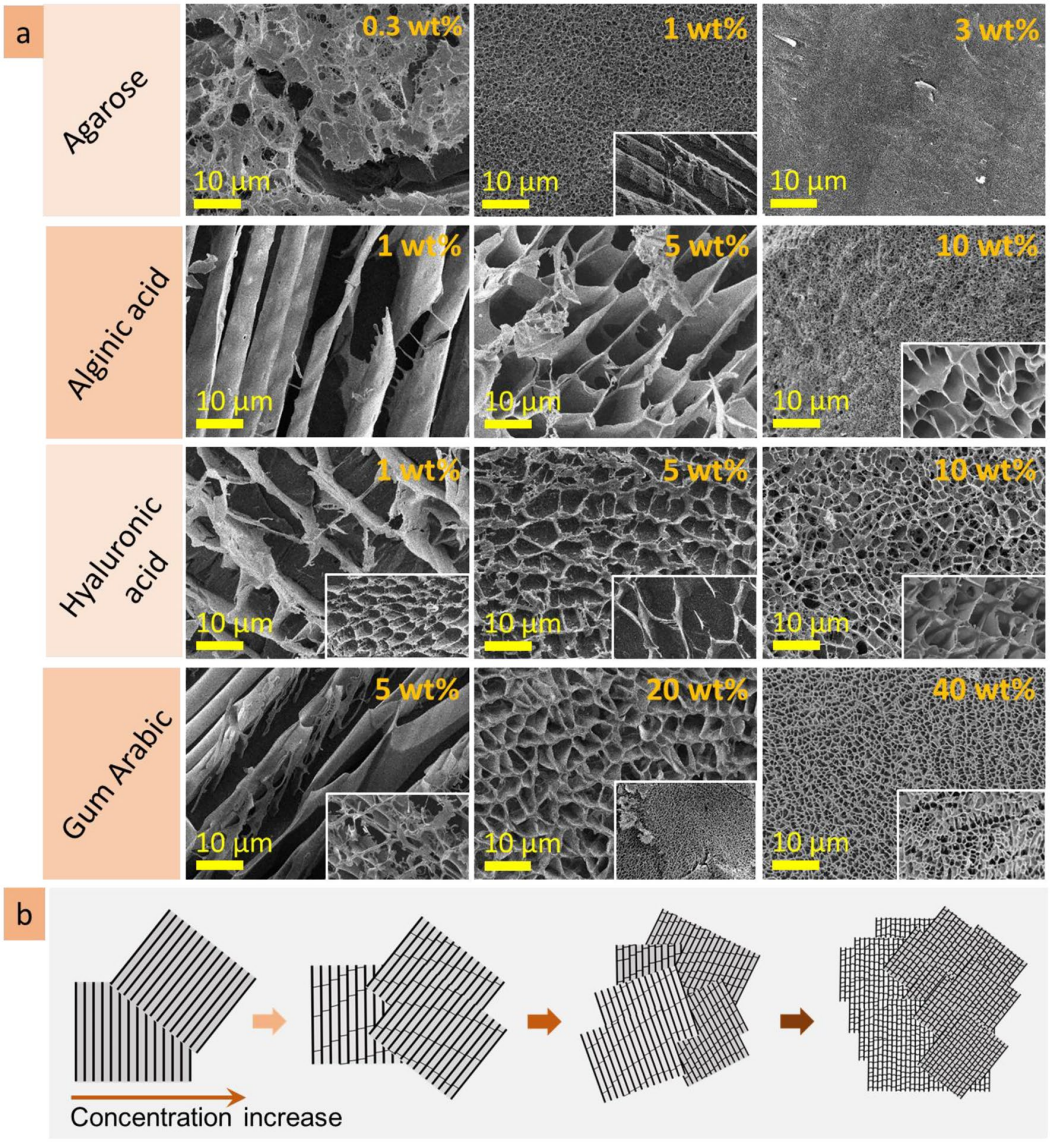
Concentration dependence of the microstructure for agarose, alginic and hyaluronic acids, gum arabic solutions: cryo-SEM images, insets have the same scale bar (**a**); scheme demonstrating transformation from lamellae, through cross-bridged lamellae to cell-like porous microstructure (**b**).

This work investigated agarose, alginic acid, hyaluronic acid and gum arabic as aqueous solutions at three concentrations each. It is shown that the microstructure of the heteropolysaccharide solutions strongly depends on concentration (Fig. 16a). At lowest concentrations, alginic, hyaluronic acids and gum arabic form predominantly lamellae structures with interlamellar distance in the range of 10 ± 2 µm. When concentration increases, the bridges appear in the lamellar structures, transforming them into porous microstructures. With an increase in concentration, the pore size decreases several times (see scheme in Fig. 16b). Agarose, which solutions tends to organise a dense gel at room temperature, being at low concentration (0.3 wt%) forms non-continuous (lumpy) porous structures with pore size in the range of 6 ± 2 µm. When concentration increases, the pore size becomes smaller (reducing up to 100–300 nm at 3 wt%). These results agree with previous findings: in particular, direct measurements of the pore size of agarose gels in water using atomic force microscopy (AFM) confirmed that the pore diameter increases when the agarose concentration decreases and that the wide pore diameter distribution narrows as the gel concentration increases^54–56^. Those experiments were specially designed to measure under aqueous conditions, allowing direct observation of the “unperturbed” gel without invasive treatment^56^.

A characteristic feature of the heteropolysaccharide solutions is the coexistence of areas with compact and permeable microstructures. The inner microstructure of the heteropolysaccharide solutions is not homogenous in scale: there are regions with smaller- or bigger-scaled structures (see insets in Fig. 16a). For agarose and hyaluronic acid, the difference in scales may be tenfold. These differences in scale would doubtfully be prescribed to insufficient mixing of the solutions (they were carefully mixed and kept at least two weeks before measurements; these observations were repeatable). If so, we can accept this observation as a feature of the heteropolysaccharide solutions. The regions with compact microstructure, hundreds of micrometres in size, may be responsible for the viscous properties of the solutions, their gelation and fluidity. They may serve as nodes that resist against the force (shear, compression, etc.) applied to the system, making it more stable, and, thus gelatinous.

**Figure 17:**
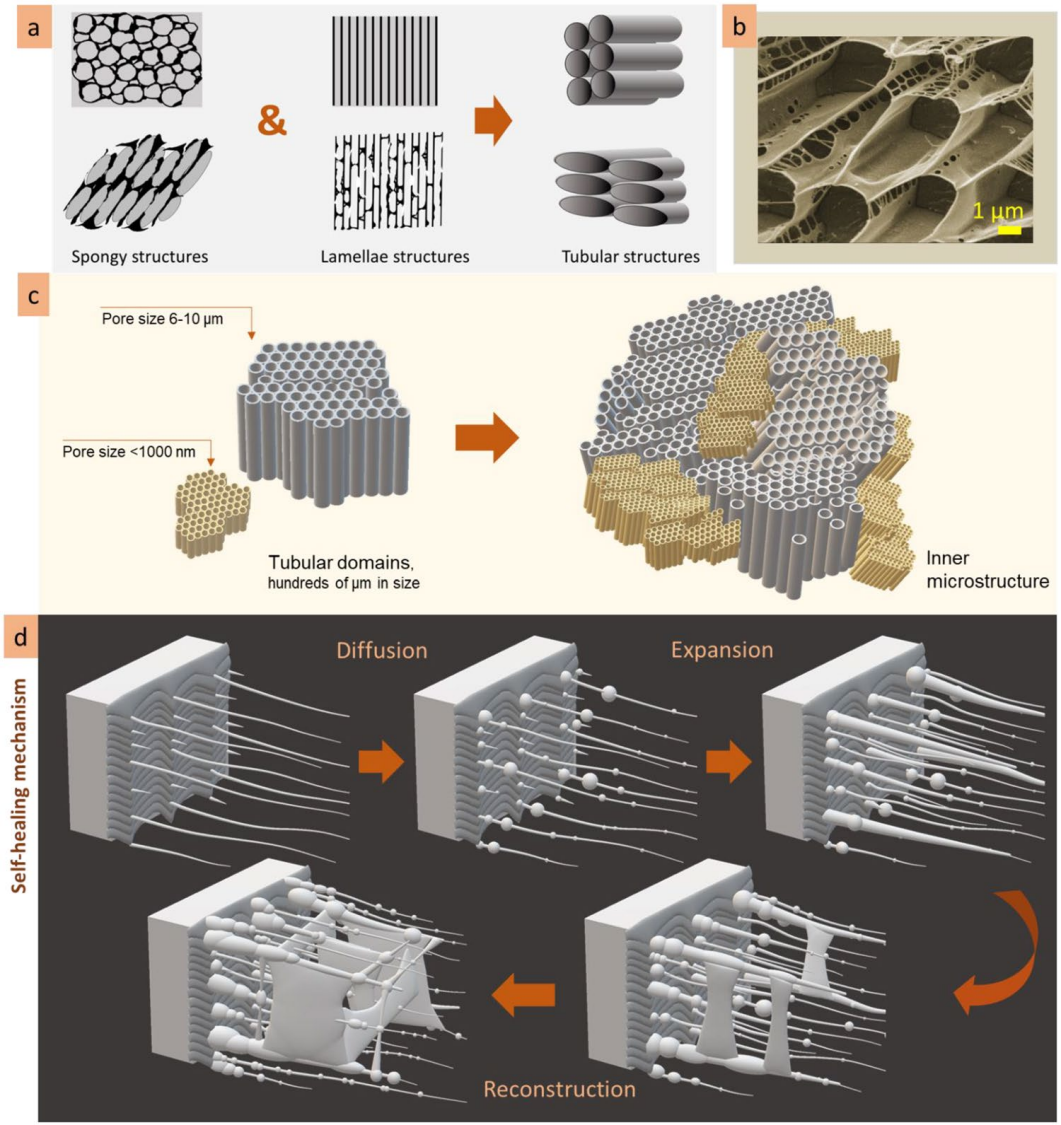
The schemes demonstrating lamellae and porous structures are presumably cross-sections of a tubular microstructure (**a**); cryo-SEM image of tubular structure of hyaluronic acid solution (**b**); microstructure model of gelatinous heteropolysaccharides: plurality of tubular domains with different microstructural scale (**c**); self-healing mechanism of the heteropolysaccharide gel after cut includes diffusion, expansion, and reconstruction stages (**d**).

The observations allowed to conclude that observed lamellae and porous microstructures are, in fact, the tubes observed at different cross-section angles (Fig. 17a,b). The walls of the tubes consist of nanofilaments, and may be perforated or continuous, depending on concentration. A tube has cross-bridges that can be filament or solid, also depending on concentration. With increasing concentration, filament cross-bridges transform into cell-like porous microstructure. The tubes are organized into three-dimensional domains hundreds of micrometers in size, in which tubes are oriented at the same directions. To summarize, the inner microstructure of the heteropolysaccharide solutions can be imagined as a plurality of three-dimensional tubular domains that have different microstructure scales—fine and coarse and are oriented at different angles (scheme in Fig. 17c).

Self-healing is among the attractive properties of gelatinous heteropolysaccharides. As a dominant compound of biological organisms, they restore functions in biological organisms, heal wounds, splice and regress damaged tissues^57^. The microscopic observations of self-healing after cut will help better understand healing processes in biological tissues. This study revealed that the self-healing of agarose and hyaluronic acid gels started from the formation of nanoscale filaments connecting the two sides of the cut (scheme in Fig. 17 d). The heteropolysaccharide molecules diffuse through these fibers into the incision site making the filaments thicker and expand the structure. Then, coalescence of fibers forms planes. Continued diffusion forms cross-bridges and cellular structures reconstructing the broken area. During 2.5 h after cutting, the development of substitutional microstructure goes from the formation of nanoscale fibres to the lamellae planes, forming irregular porous structures. The substituting microstructure is bigger in scale and has irregularities in shape and size. The microstructure will probably become homogeneous during the next hours of self-healing because of the continuous diffusion processes. The diffusion of molecules into the incision site goes from the stripe region (approx. 50 µm in width) along the cut border; the microstructure in this region became bigger in scale in time due to the local decrease of concentration. The formation of the substituting microstructure correlates with the concentration-dependent changes in the microstructures. Indeed, the substitutional microstructure develops from filaments to lamellae, then lamellae with bridges, and finally, an irregular porous structure is formed. Similar transformations in microstructure were observed when the concentration of the solutions increased. The self-healing process is based on the diffusion of heteropolysaccharide molecules to the cut site that increases low local concentration and allows to formation of a substitutional microstructure.

Investigation of the reorganisational abilities of heteropolysaccharide solutions showed that their microstructure responds to the substrate topology's roughness by forming a transitional layer. The width, pattern and scale of the transitional layer depend on the size of topological elements (pores, indentations, holes, etc.) of the surface. The bigger the roughness of the substrate, the wider the transitional layer and the bigger gradient in scale.

## Conclusions

In this study, the microstructures of the heteropolysaccharide solutions were investigated by means of two microscopy techniques: cryogenic scanning electron microscopy (cryo-SEM), which produces an image of the frozen samples due to emission of the secondary electrons, and confocal laser scanning microscopy (CLSM) that utilize fluorescence properties for sample visualization. Both techniques demonstrated the significant differences between microstructure layers of the heteropolysaccharide solutions. The following microstructure layers have been differentiated: surface, subsurface, inner, transitional, and expansive layers.

- It is shown that the microstructure’s patterns and scale of the heteropolysaccharide solutions strongly depend on concentration. With an increase in concentration, the microstructure pattern transforms from lamellae to cell-like structures, and scale decreases several times.
- The inner microstructure of the heteropolysaccharide solutions is not homogenous: there are regions with smaller- or bigger-scaled structures. It is supposed that the regions with finer microstructure are responsible for the viscous properties of the solutions, their gelation and fluidity.
- Investigation of the self-healing mechanism showed that this process goes through the stages (diffusion, expansion, reconstruction) corelated with the increase of concentration: from fibres to lamellae, then interconnected lamellae, and finally results in an irregular porous cell-like structure. Substituting microstructure is usually bigger in scale and has irregularities in shape and size.
- Investigation of the self-reorganisational abilities of the heteropolysaccharide solutions showed that their microstructure responds to the roughness of the substrate topology by the formation of the transitional layer. It was shown that the higher the roughness of the substrate, the wider the transitional layer, and the bigger gradient in its scale.

This work contributes to understanding the microstructural peculiarities of heteropolysaccharide solutions looking at them through a prism of supramolecular, micro- and nano-levels. The peculiarities of the heteropolysaccharide microstructures allow to better understand, from a structural perspective, their properties and prevalence in the animal and plant worlds, as well as a potential for biomedical and technological applications.

## Materials and methods

### Materials

The analytical grade reagents of agarose (for routine use, molecular biology tested) (CAS no. 9012-36-6), alginic acid sodium salt from brown algae medium viscosity (≥ 2.000 cP, 2%, 25 °C) (CAS no. 9006–38-3), hyaluronic acid sodium salt from *Streptococcus equi* (mol. wt ~ 1.5–1.8 × 10^6^ Da) (CAS no. 9067-32-7), gum arabic from Acacia tree (spray dried acacia gum) (CAS no. 9000-01-5), fluorescence dye Atto 550 NHS ester (MDL no. MFCD05865403), dimethyl sulfoxide (DMSO) (CAS no. 67-68-5) were purchased from Merck (Sigma-Aldrich) and used as received. Ultrapure water (resistivity > 17 MΩcm) from a GZY-P10 water system was used throughout the experiments. Glass slides 24 × 50 mm (Menzel Gläser) and 18 × 18 mm (Zeiss), and rubber gasket (o-ring, 13 × 1 mm) were used for confocal LSM measurements.

In this study, agarose (0.3, 1, 3 wt%), alginic acid (1, 5, 10 wt%), hyaluronic acid (1, 5, 10 wt%), and gum arabic (5, 20, 40 wt%) solutions with addition of fluorescence dye Atto 550 NHS ester were used. To prepare the heteropolysaccharide solutions with fluorescence dye Atto 550 NHS ester, 4 µl of the dye stock solution (1 mg/100 µl DMSO) was diluted in 1 ml of water. Then, a weighted amount of the heteropolysaccharide powder was added. Hyaluronic acid and gum arabic were solved at room temperature, whereas agarose and alginic acid solutions were kept in a thermoresistant glass in a microwave oven for a few minutes, avoiding boiling. Freshly prepared solutions were stored in the dark place at 4 °C at least 48 h before measurements. The Atto 550 NHS ester concentration in the heteropolysaccharide solutions was 40 µg/ml.

Fourier-transform infrared (FTIR) spectroscopy measurements of the heteropolysaccharide powdered compounds were performed using a FT/IR-4700 spectrometer (JASCO) equipped with a ATR PRO ONE mode (in the range of 400–4000 cm^−1^, room temperature). Morphological investigations of the heteropolysaccharide powdered compounds were performed using JEOL 7001 F Scanning Electron Microscope (SEM) (accelerated voltage 10 kV, gold spattering).

*Cryo-SEM measurements* were performed using a scanning electron microscope JEOL 7001F equipped with PP3000T Cryo System manufactured by Quorum Technologies. The PP3000T Cryo System utilizes a nitrogen slush plunge-freezing method for rapid sample freezing (liquid nitrogen freezes at − 210 °C). Following the protocol (PP3000T User Manual v.1.4), rapid specimen freezing was performed at the workstation (trademarked PrepDek), which includes slusher pots for specimen preparation and manipulation and the control electronics. Liquid samples were put as a drop (~ 30 µl), and agar gel as a piece into a hole of specimen stub so that it protrudes above the surface. A frozen specimen was fractured in the preparation chamber. Then, sublimation (ice etching) was performed at − 90 °C for 45 min, followed by plasma sputtering. Plasma sputter coating was performed using Pt target. The temperature of the cold stage in the electron microscope was maintained at ‒190 ± 2 °C (vacuum level ~ 2.6·10^–5^ Pa). Secondary electron images of the frozen specimen were acquired at 5 keV.

### Confocal LSM measurements

Fluorescence imaging of the heteropolysaccharide solutions was performed at room temperature using confocal LSM Zeiss LSM 780 equipped with a femtosecond tuneable infrared laser for two-photon excitation. The specimen (30 µl or 4 × 4 mm piece of gel) was placed on a glass slide (24 × 50 mm), with a rubber gasket (o-ring 13 × 1 mm) on top, inside the rubber gasket (to keep the specimen volumetric during the measurement; thickness of a rubber pad ≈ thickness of specimen) and covered by another glass slide (to reduce water evaporation during measurements). The measurements of the samples were performed using objective C-Apochromat 40x/1.20 W Corr M27 (numerical aperture 1.2). For the agarose solutions imaging, laser excitation wavelength was 561 nm with laser power in the range of 0.04–0.07% of nominal laser power, and emission was collected in the 573–735 nm range. For hyaluronic, alginic acids and gum arabic, laser excitation wavelength was 405 nm with laser power 0.01%, emission waves were collected in the range of 569–740 nm. Gain 830, 1 AU, and line average mode were set for image recording. The concentration of the fluorescence dye Atto 550 NHS in the heteropolysaccharide solutions was 40 µg/ml*.*

*Self-healing mechanism* was investigated using gelatinous heteropolysaccharide solutions that keep their shape when cut (hyaluronic acid 10 wt%, agarose 1 wt% and 3 wt%) by cryo-SEM and CLSM techniques. For cryo-SEM measurements, a piece of gelatinous sample was fixed on a specimen stub and cut with a scalpel. The incision site was investigated at two intervals: a few seconds after cutting and 2.5 h later, keeping the sample in a humid atmosphere at room temperature (21 °C). For CLSM measurements, a piece of jelly heteropolysaccharide solution was cut by a scalpel and put on a glass slide. Imaging of the incision site was performed during 2–3 h after cutting. Fig. S1 presents photos of the specimens for CLSM and cryo-SEM measurements.

*Reorganisational ability* of the heteropolysaccharide solutions was investigated using the specially prepared porous substrate. For the preparation of the porous substrate, an aluminium specimen stub was sonicated in hydrochloric acid (0.1 M) solution for 30 min, then washed with water and ethanol. For the cryo-SEM measurements, a drop of the heteropolysaccharide solution was exposed on the acid-treated specimen stub for 2 h 40 min, at 21 °C and increased humidity. Control experiments were performed using non-acid-treated specimen stubs (as received from the manufacturer) following the same protocol of the experiment.

### Supplementary Information

Supplementary Information.

## Data Availability

The datasets used and/or analysed during the current study available from the corresponding author on reasonable request.
