# Supplementary material for: Layered complexity, reorganisational ability and self-healing mechanisms of heteropolysaccharide solutions
Source: Sci Rep. 2024 Jun 17;14:13957. doi: 10.1038/s41598-024-64873-0 (PMC11183217; doi:10.1038/s41598-024-64873-0)
Supplement: Supplementary file 1 — Supplementary Information. [file 41598_2024_64873_MOESM1_ESM.docx]

Layered Complexity, Reorganisational Ability and Self-Healing Mechanisms of Heteropolysaccharide Solutions

*Olena Ivashchenko*

NanoBioMedical Centre, Adam Mickiewicz University, 61-614, Poznań, Poland

**SUPPLEMENTAL DATA**


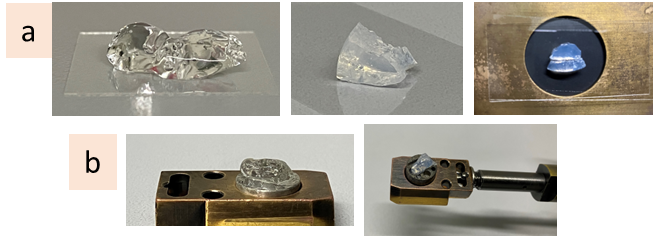


**Figure S1.** Hyaluronic acid and agarose solutions used for CLSM (a), and cryo-SEM measurements (b).

**
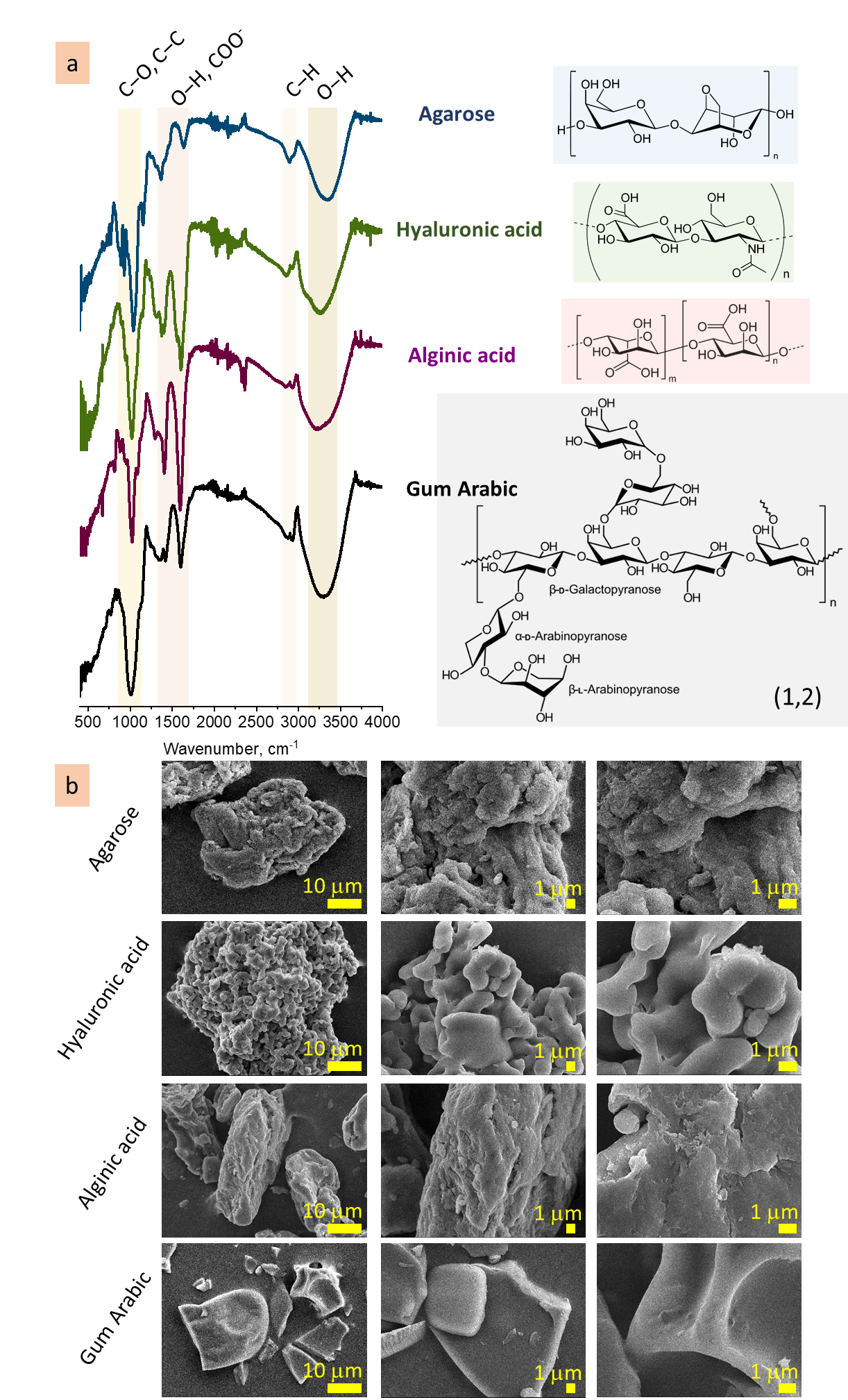
**

**Figure S2.** FTIR spectra and structural formulas of agarose, alginic acid, hyaluronic acid and gum Arabic (a),^1,2^SEM images of agarose, alginic acid, hyaluronic acid and gum Arabic powders as received (b).


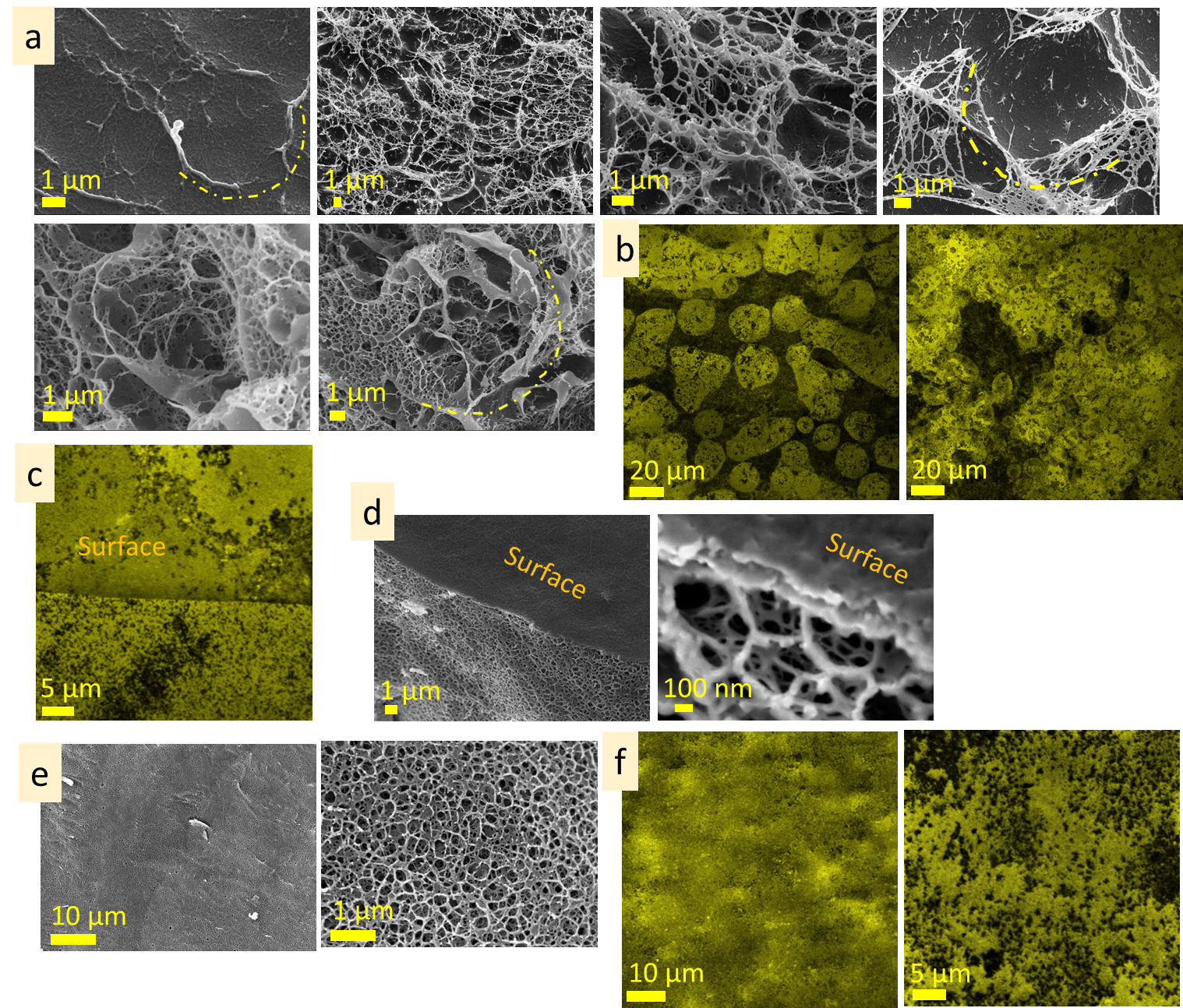


**Figure S3.** Microstructure of agarose (3 wt%): cryo-SEM and CLSM images of surface (a, cryo-SEM; b, CLSM), under surface layers (c, CLSM; d, cryo-SEM), and inner microstructure (e, cryo-SEM; f, CLSM).


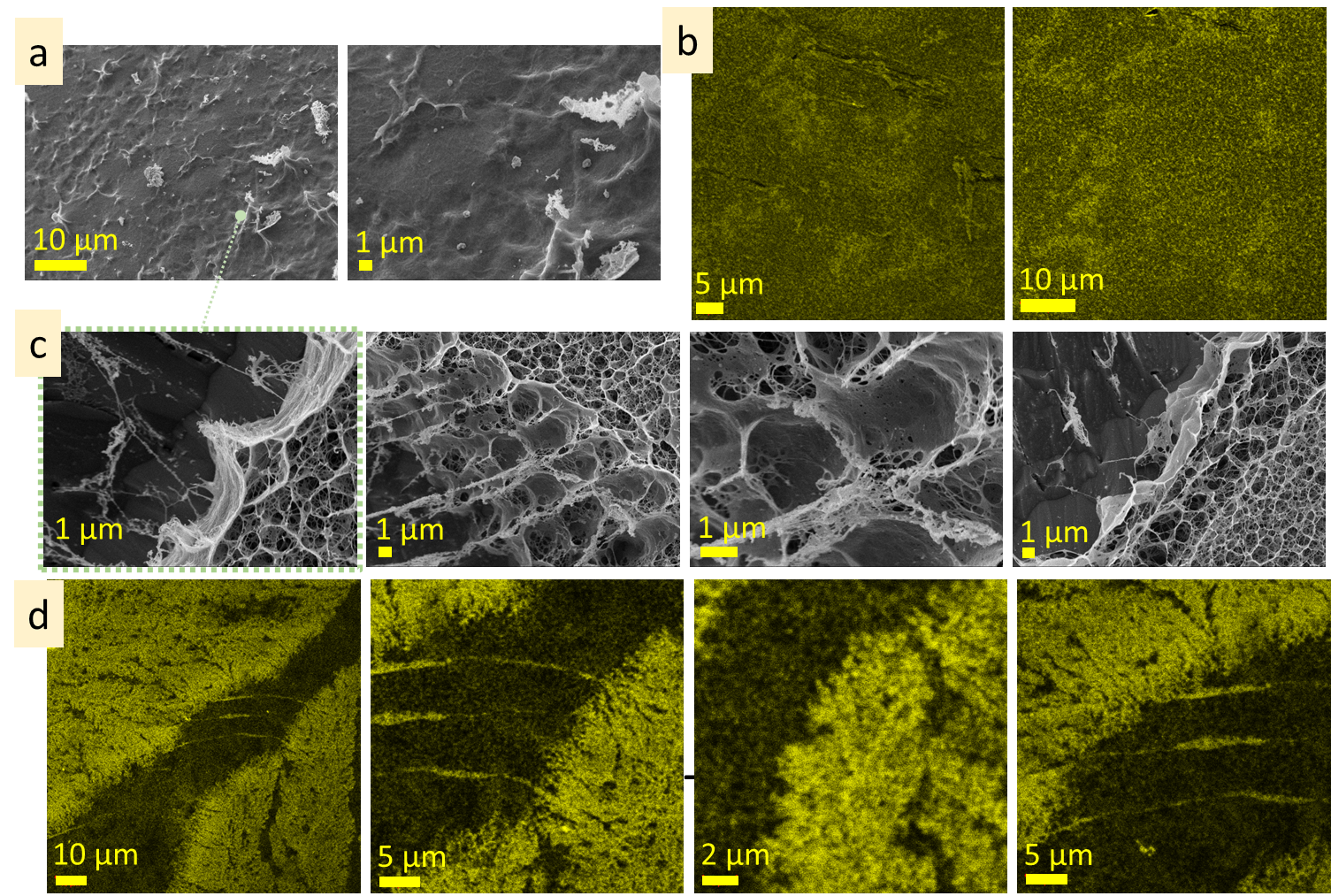


**Figure S4.** Microstructure of agarose (1 wt%): surface (a, cryo-SEM; b, CLSM), inner (c, cryo-SEM; d, CLSM).


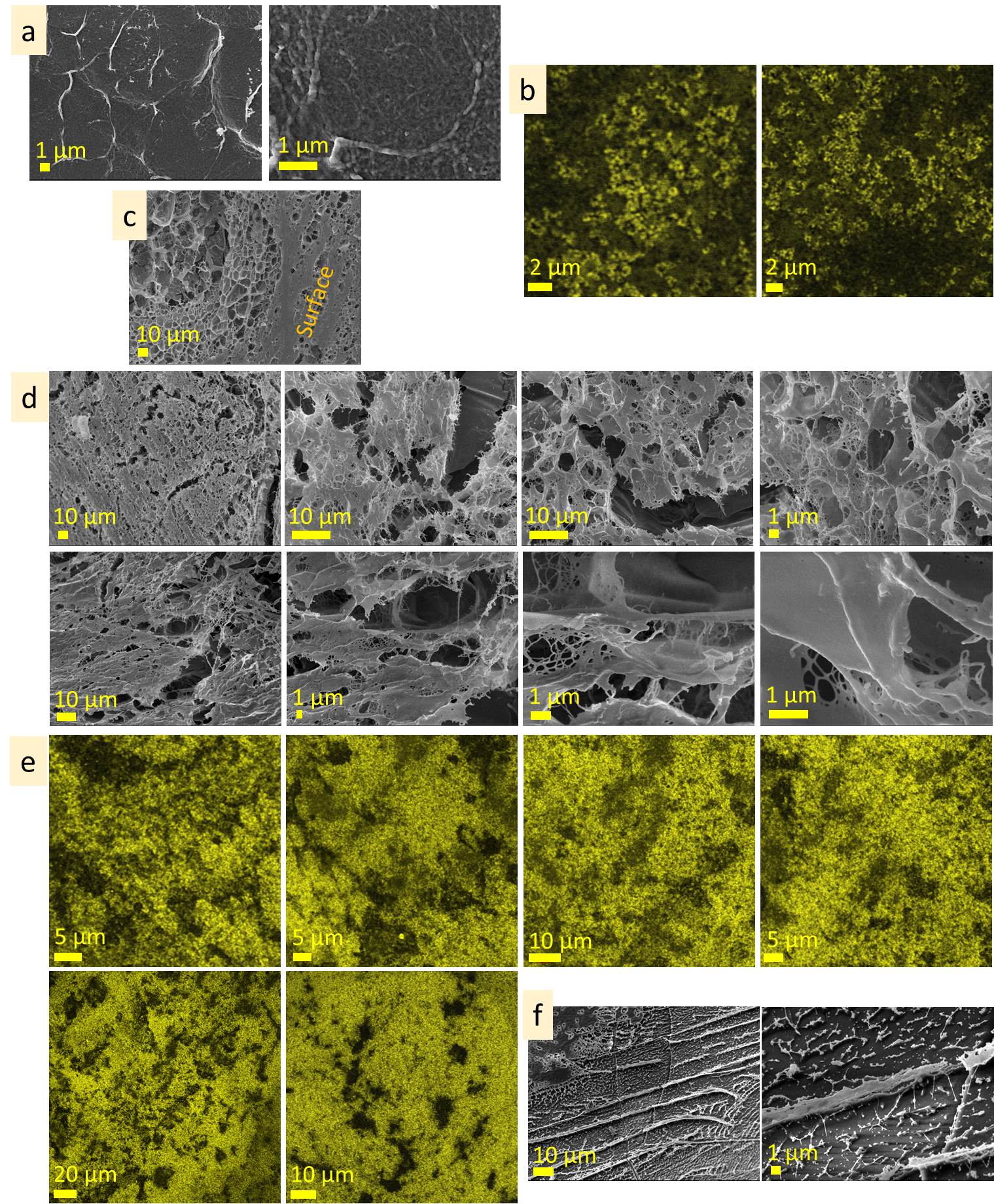


**Figure S5.** Microstructure of agarose (0.3 wt%): surface (a, cryo-SEM; b, CLSM), under surface (c, cryo-SEM), inner (d, cryo-SEM; e, CLSM), expansive (f, cryo-SEM) layers.


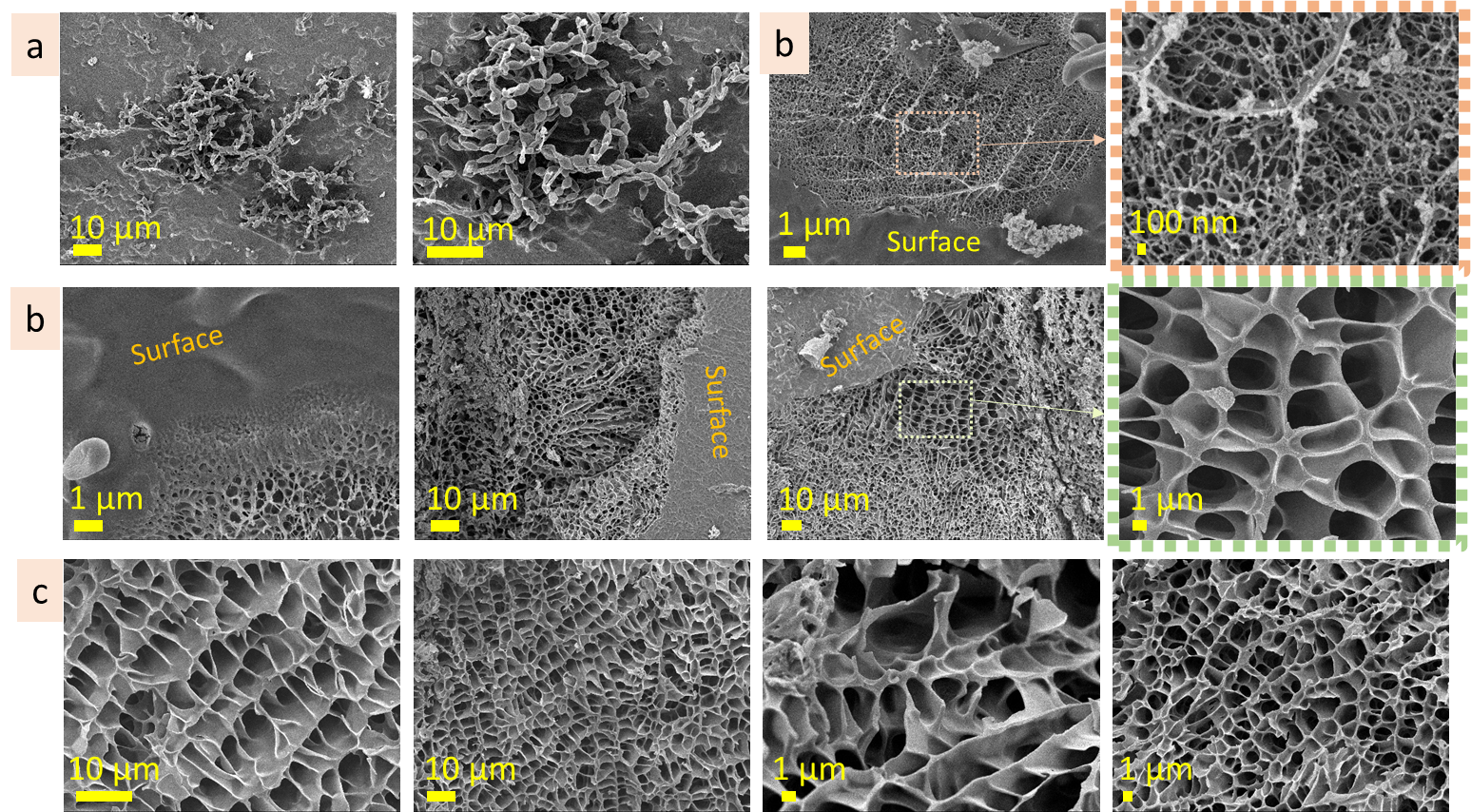


**Figure S6.** Microstructure of alginic acid (10 wt%): surface (a, cryo-SEM), under surface (b, cryo-SEM), inner (c, cryo-SEM) layers.


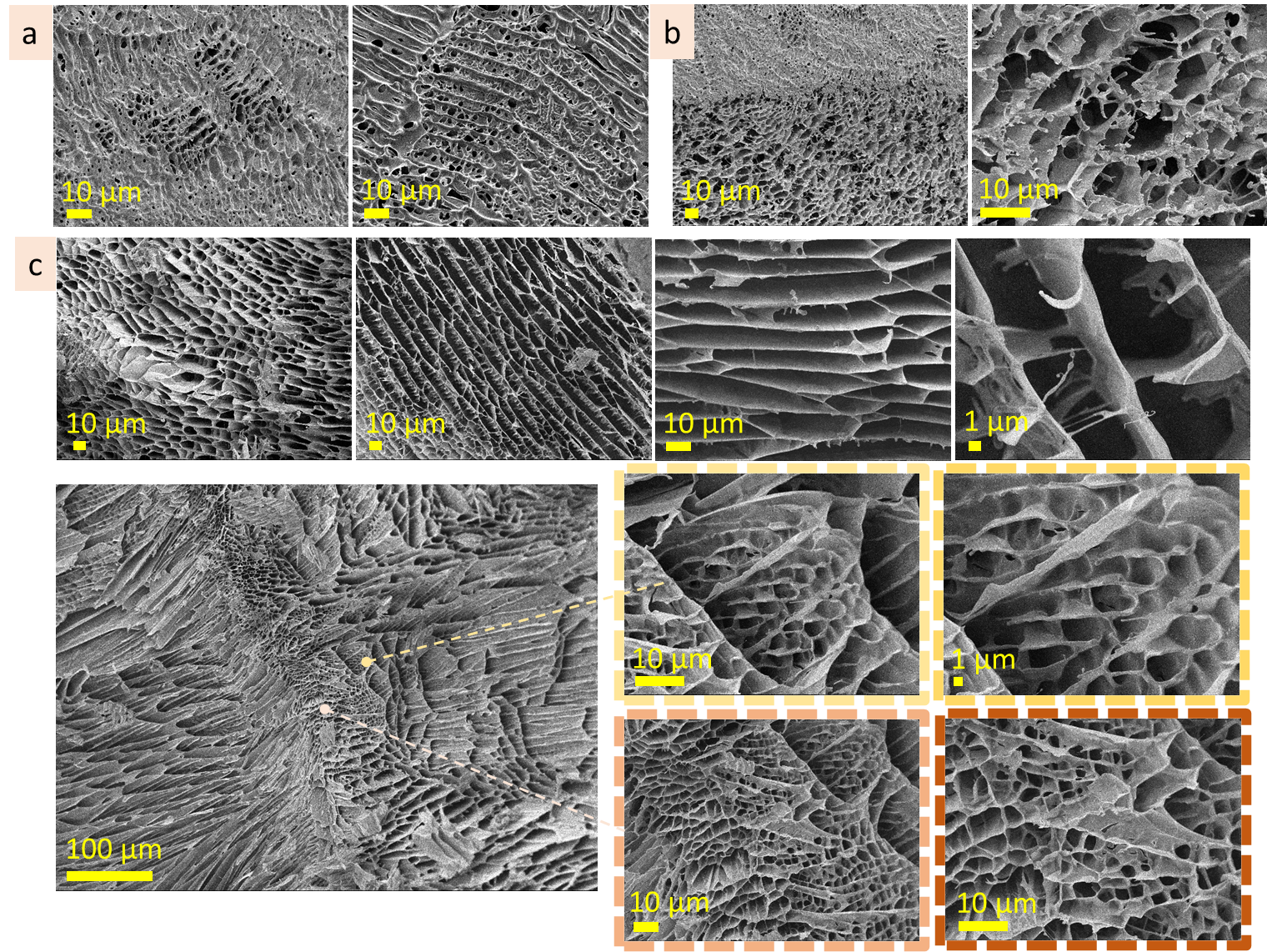


**Figure S7.** Microstructure of alginic acid (5 wt%): surface (a, cryo-SEM), under surface (b, cryo-SEM), inner (c, cryo-SEM) layers.


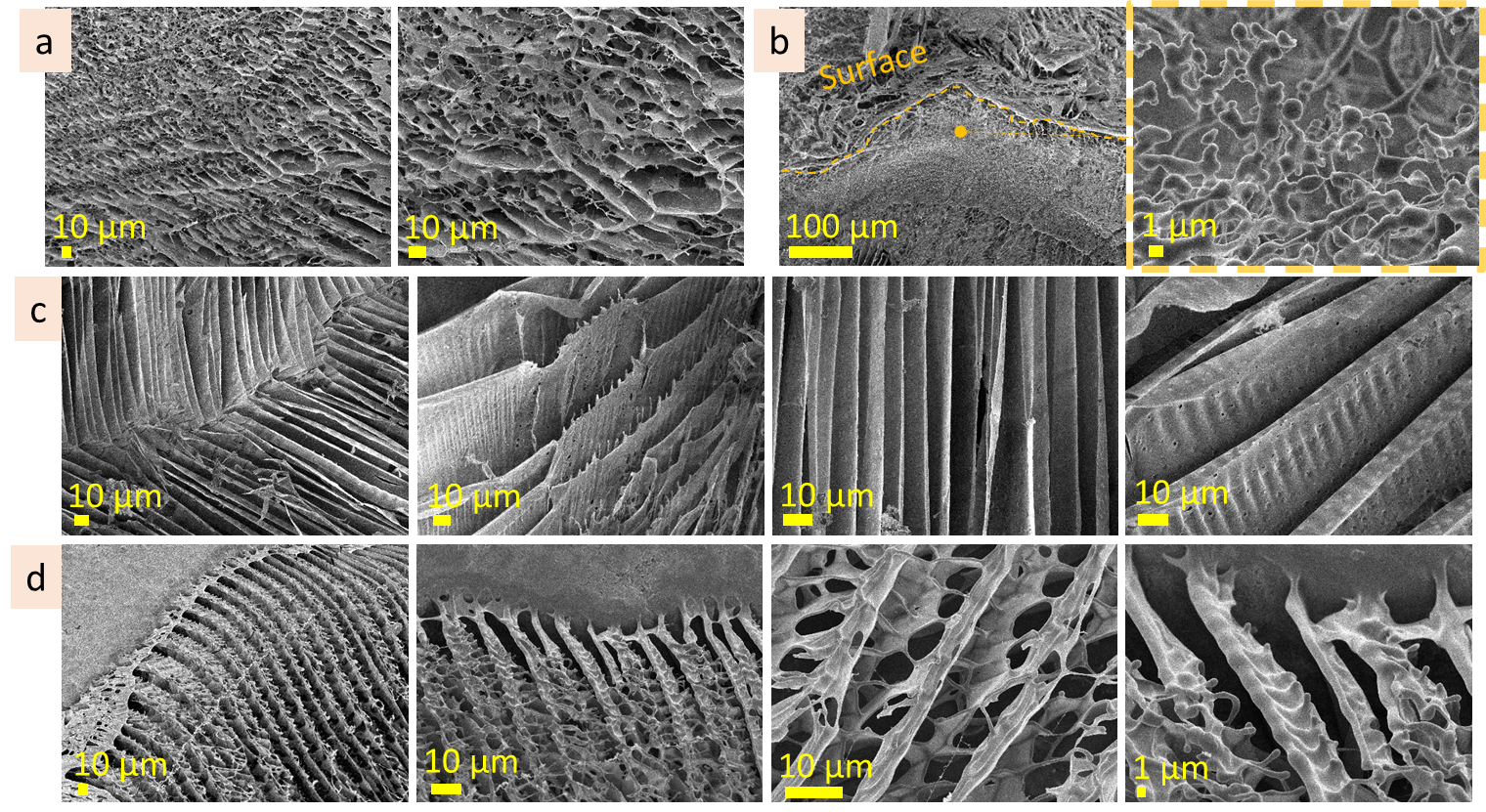


**Figure S8.** Microstructure of alginic acid (1 wt%): surface (a, cryo-SEM), under surface (b, cryo-SEM), inner (c, cryo-SEM), expansive (d, cryo-SEM) layers.


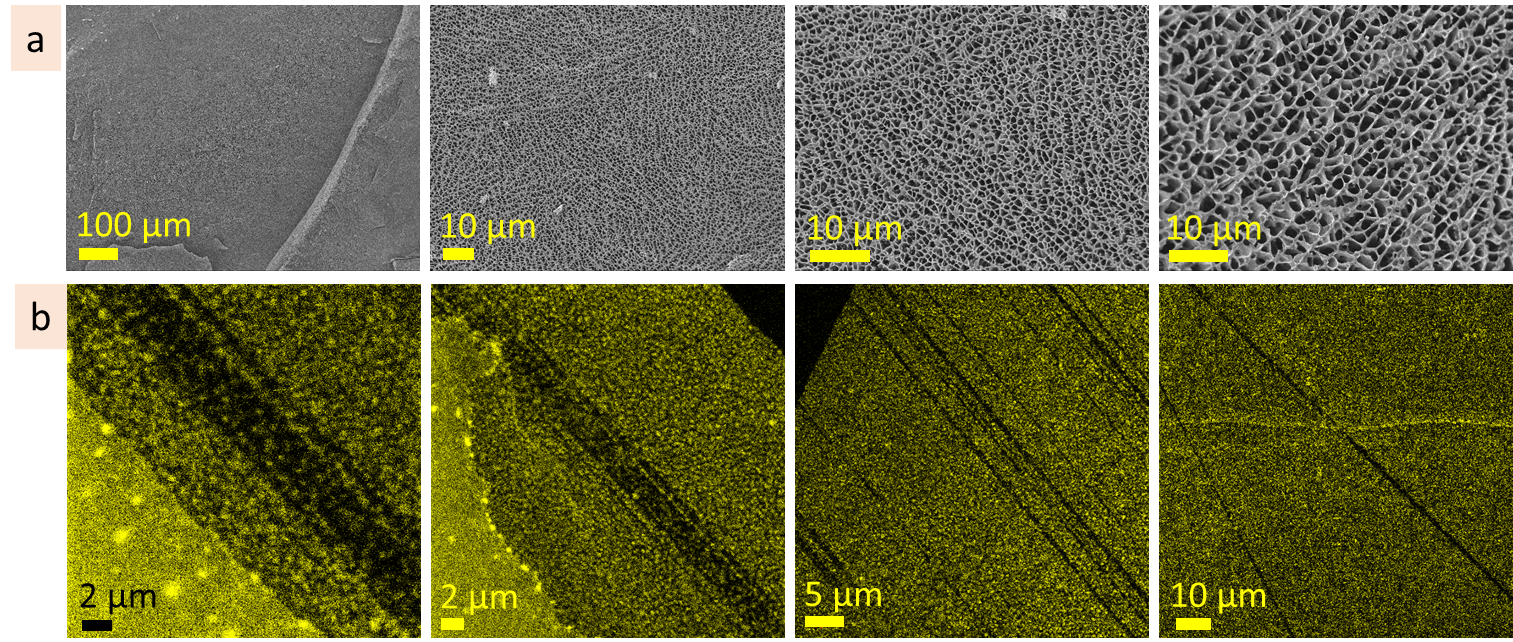


**Figure S9.** Microstructure of gum Arabic (40 wt%): inner (a, cryo-SEM), expansive (c, CLSM) layers.


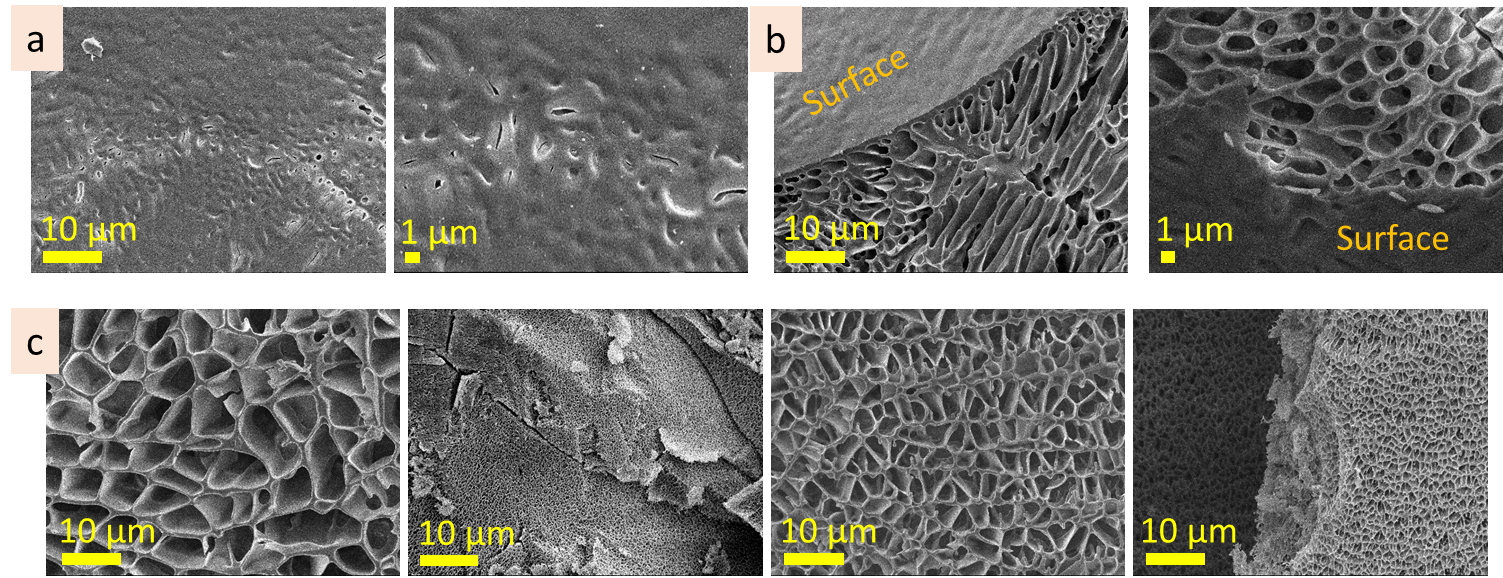


**Figure S10**. Microstructure of gum Arabic (20 wt%): surface (a, cryo-SEM), under surface (b, cryo-SEM), inner (c, cryo-SEM).


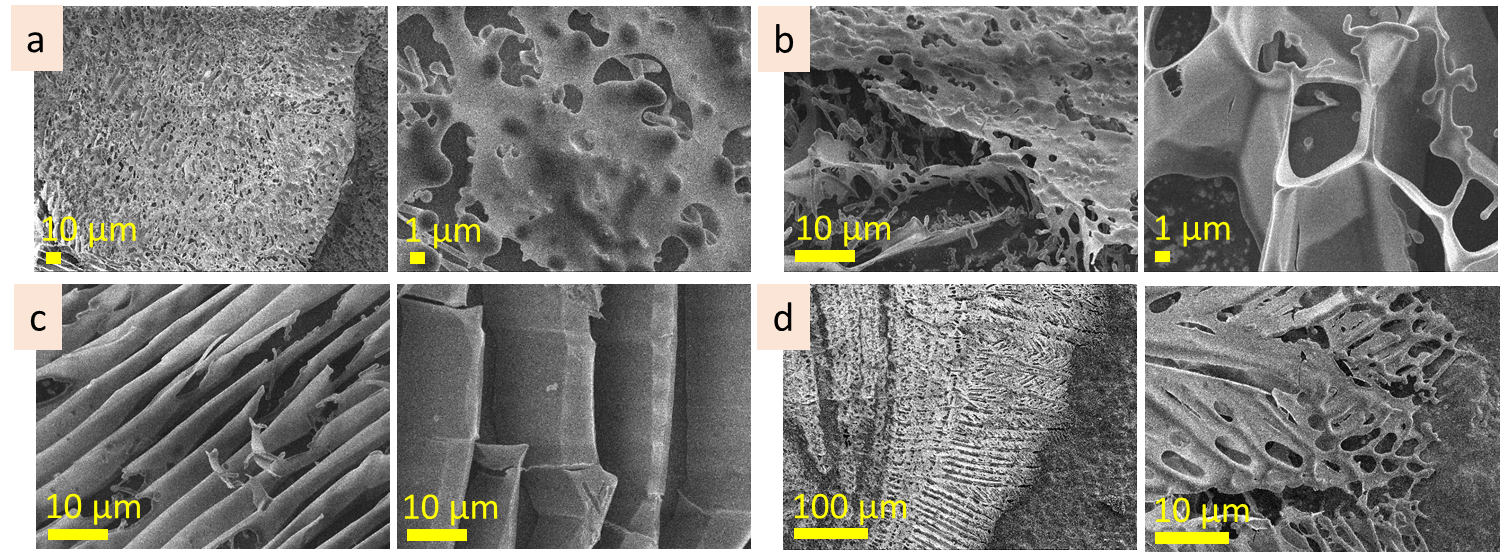


**Figure S11**. Microstructure of gum Arabic (5 wt%): surface (a, cryo-SEM), under surface (b, cryo-SEM), inner (c, cryo-SEM) layers, and expansive (d, cryo-SEM) structures.


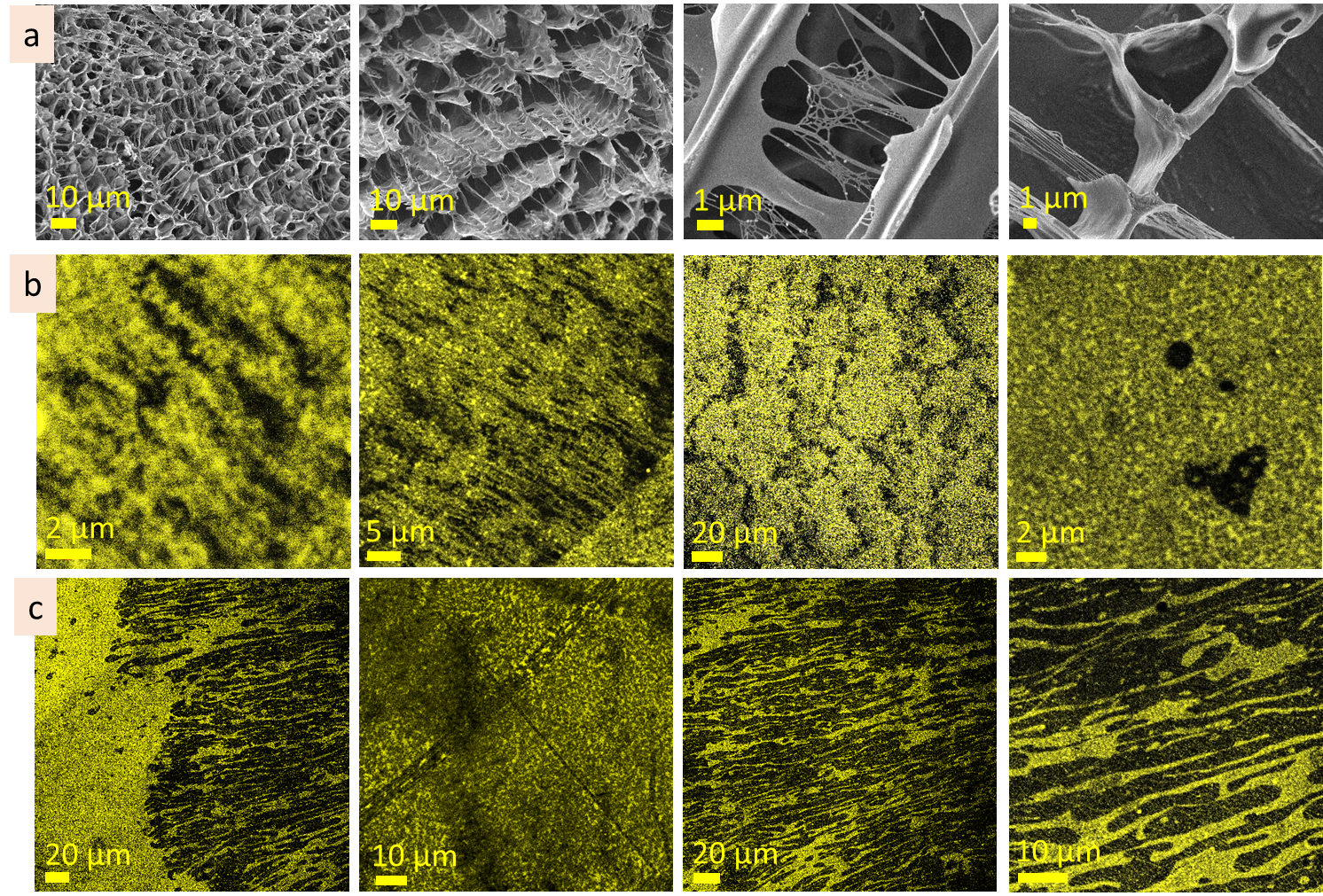


**Figure S12.** Microstructure of hyaluronic acid (10 wt%): inner (a, cryo-SEM; b, CLSM), expansive structures (c, CLSM).


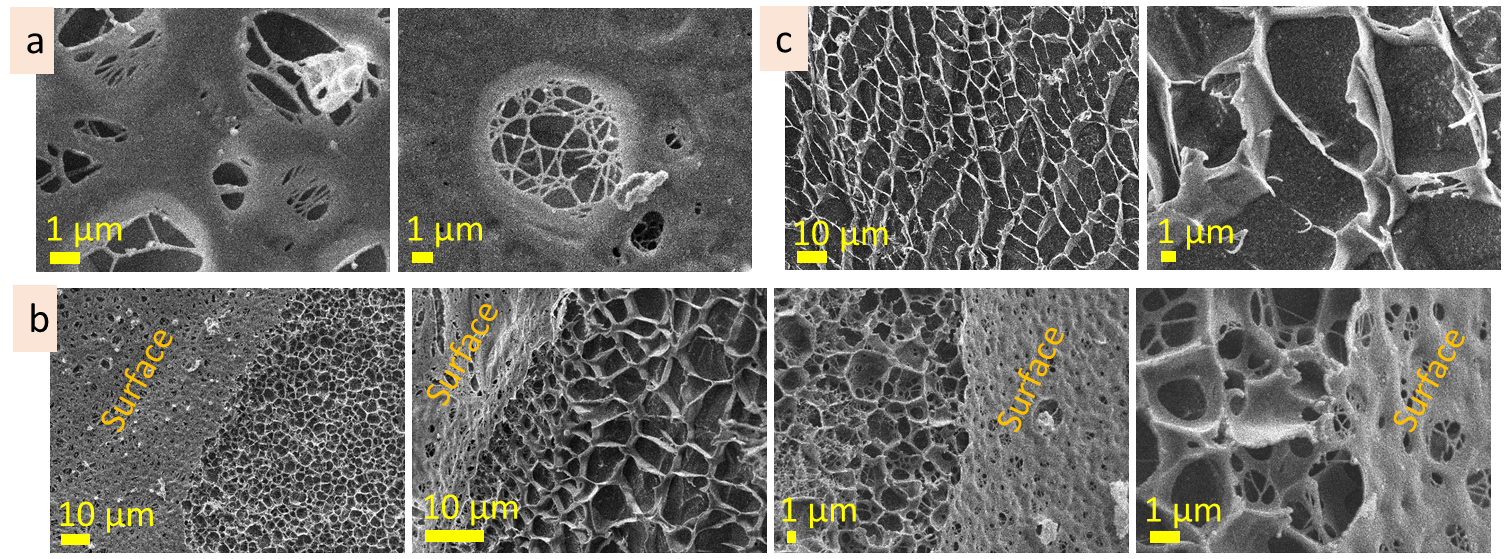


**Figure S13.** Microstructure of hyaluronic acid (5 wt%): surface (a, cryo-SEM), under surface (b, cryo-SEM), inner (c, cryo-SEM) layers.


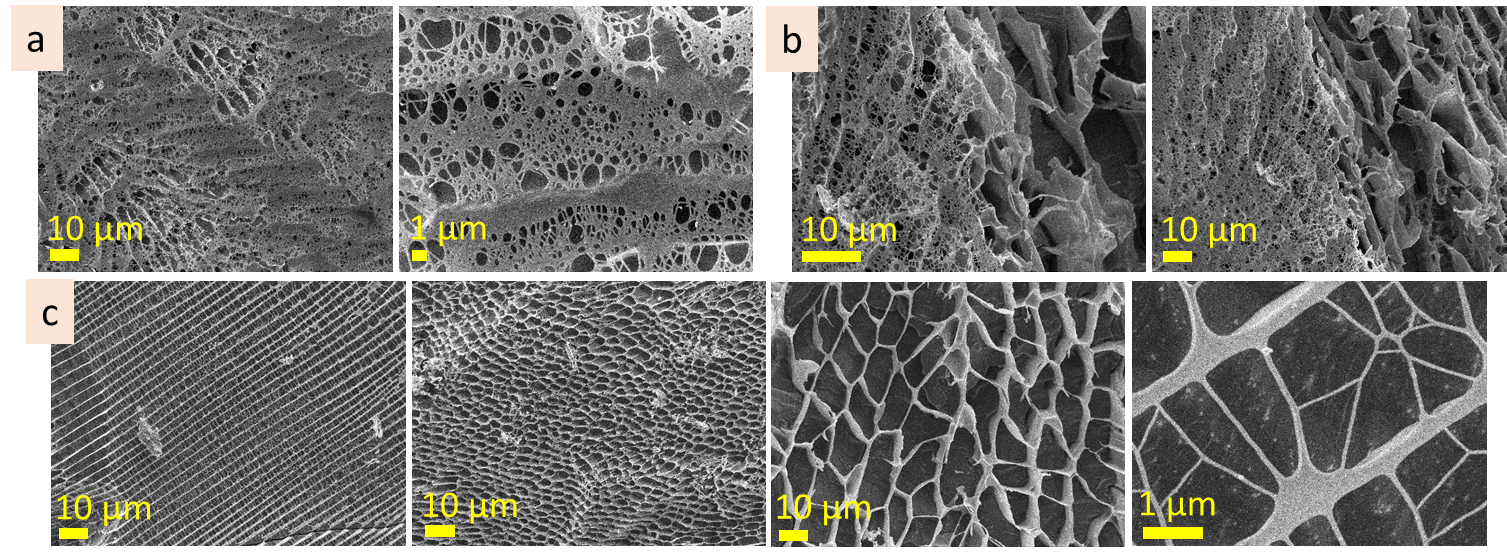


**Figure S14.** Microstructure of hyaluronic acid (1 wt%): surface (a, cryo-SEM), under surface (b, cryo-SEM), inner (c, cryo-SEM) layers.


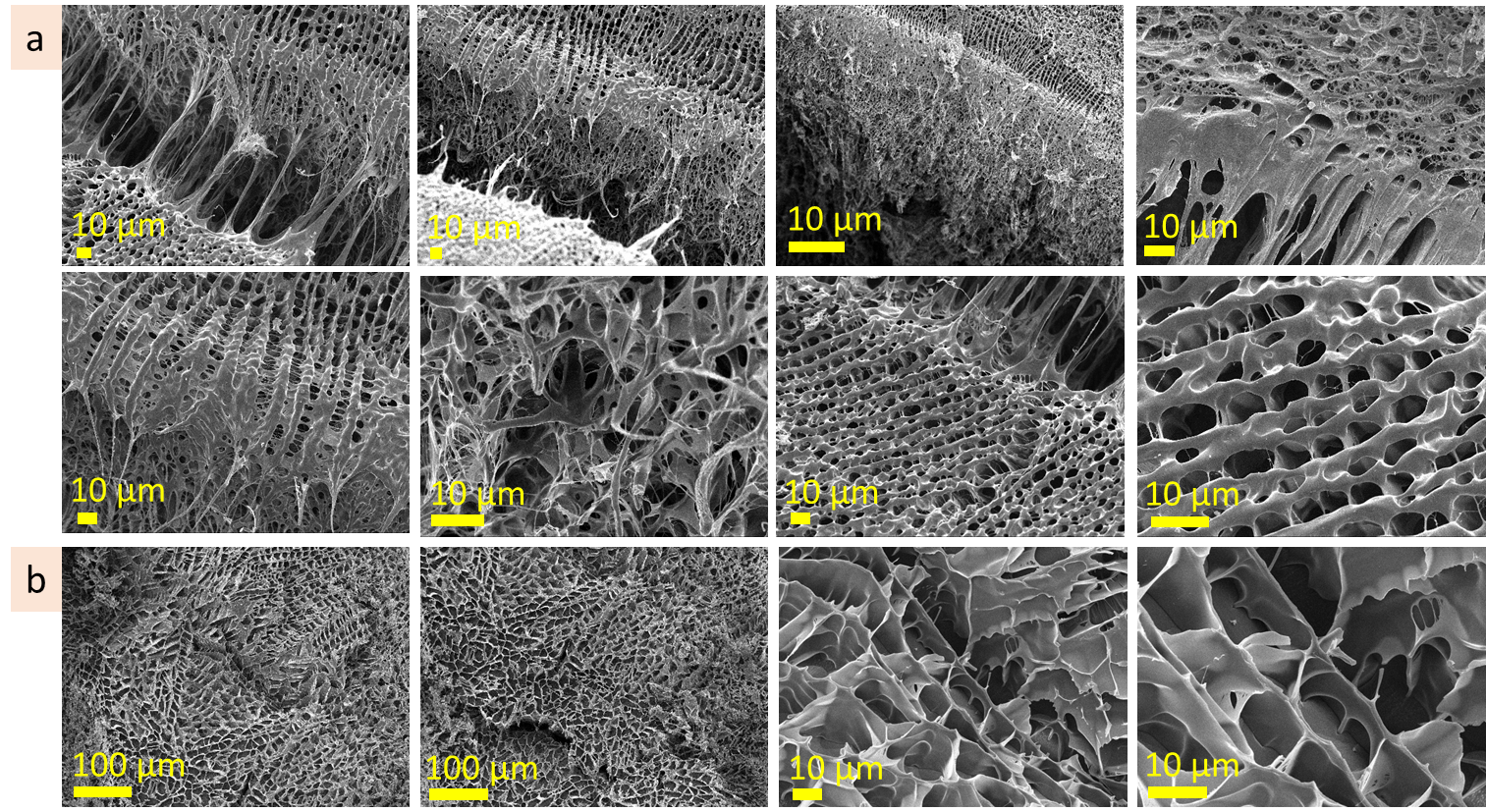


**Figure S15.** Self-healing: the incision site at hyaluronic acid (10 wt%) cut a few seconds before freezing (a), and 3h before freezing (b), cryo-SEM images.


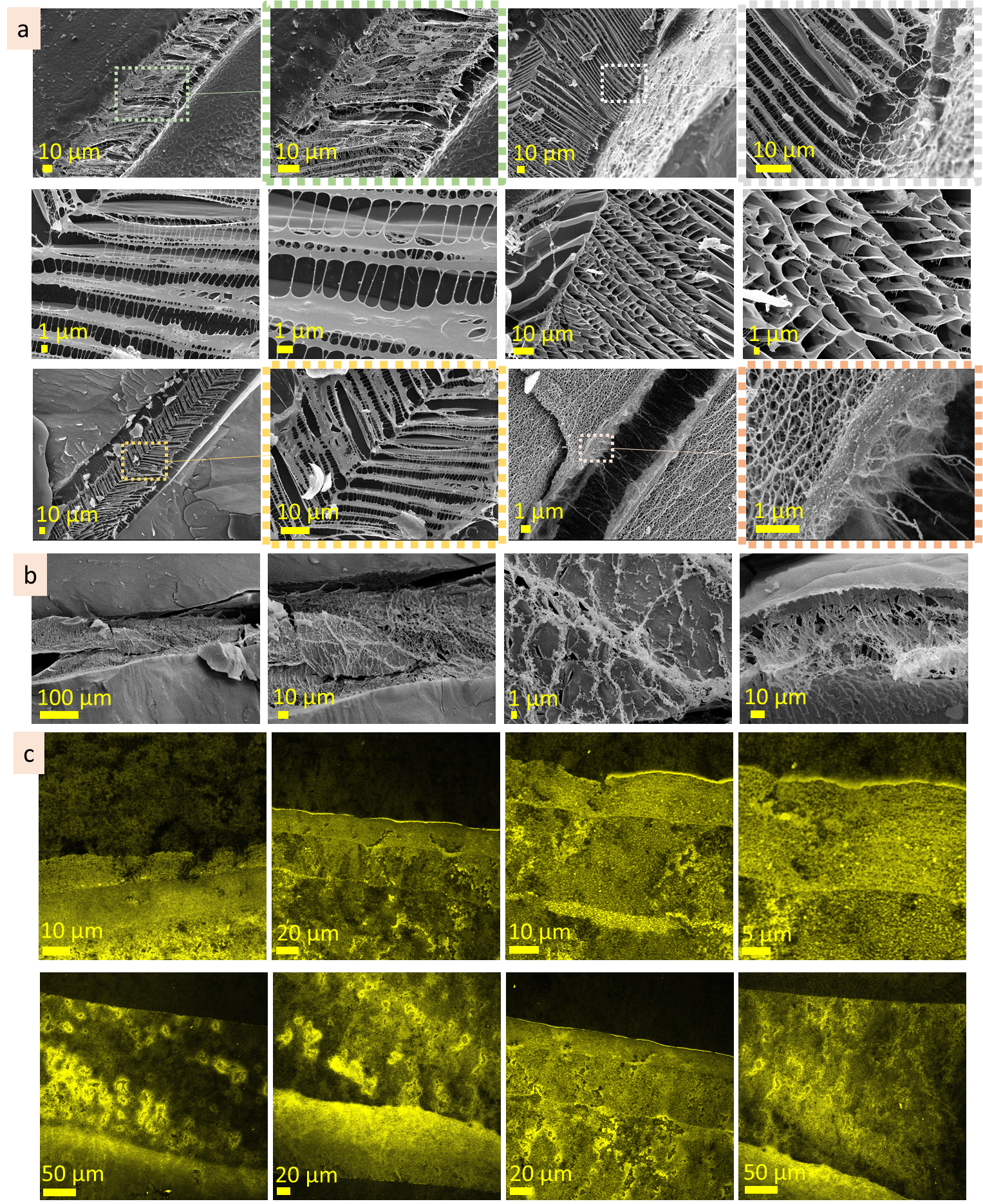


**Figure S16.** Self-healing: the incision site at agarose (3 wt%): a few seconds after cutting (a, cryo-SEM), 2.5 h after cutting (b, cryo-SEM), CLSM images (c).


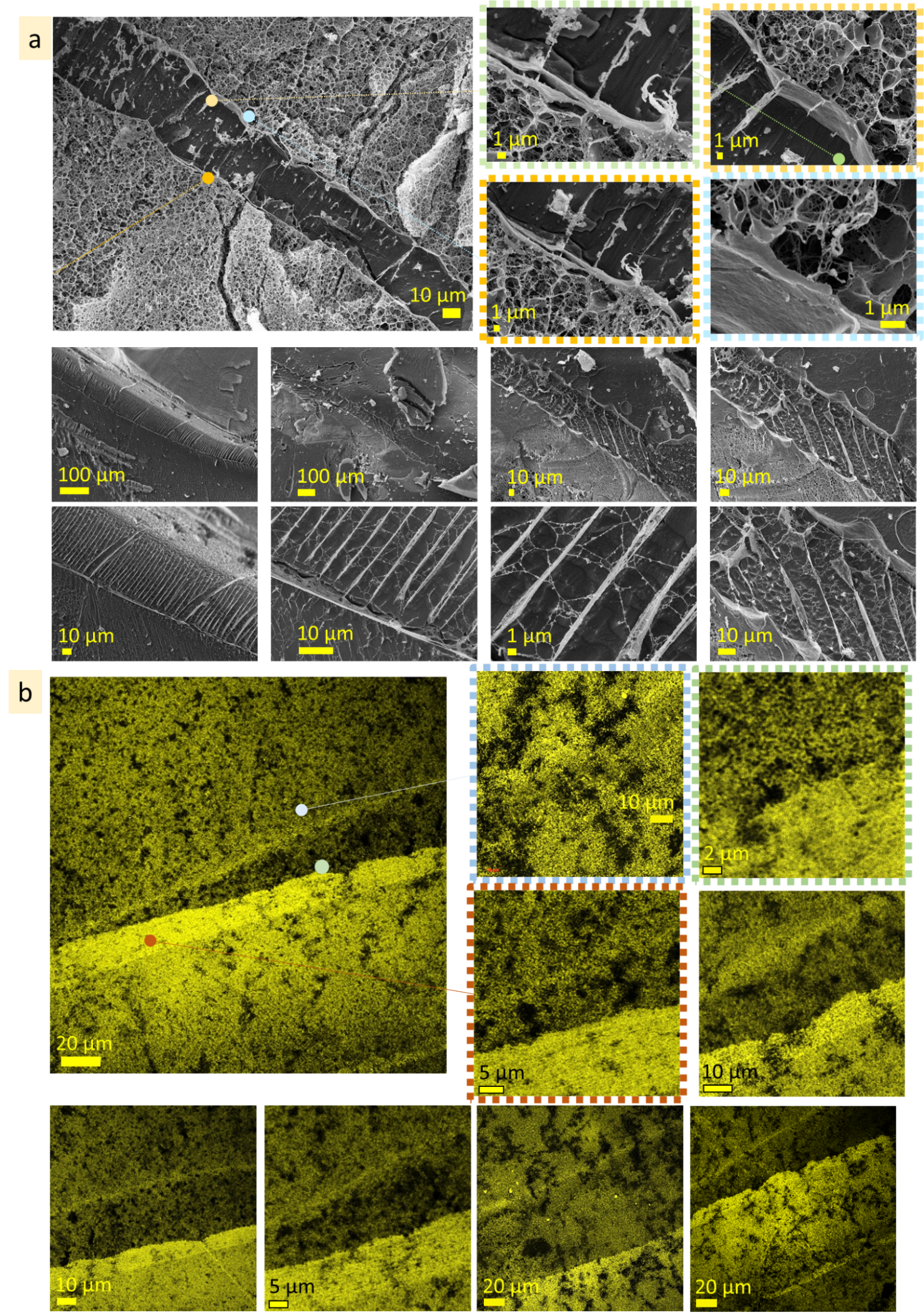


**Figure S17.** Self-healing: the incision site at agarose (1 wt%) (a, cut seconds before freezing, cryo-SEM; b, CLSM).


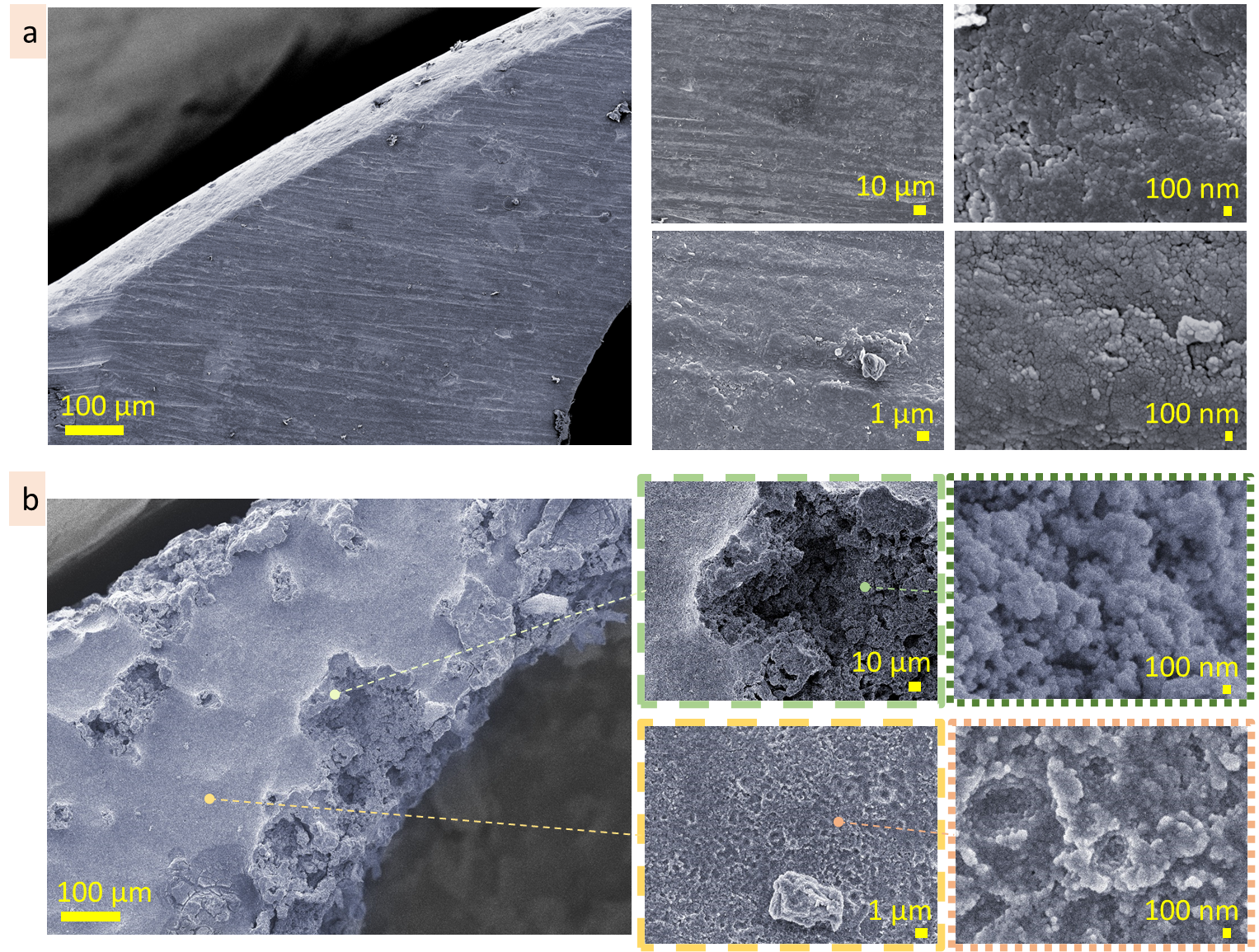


**Figure S18.** Micro-to-nano porous substrate (acid-treated aluminium specimen stub) used for polysaccharide solutions exposition (SEM, coloured images).


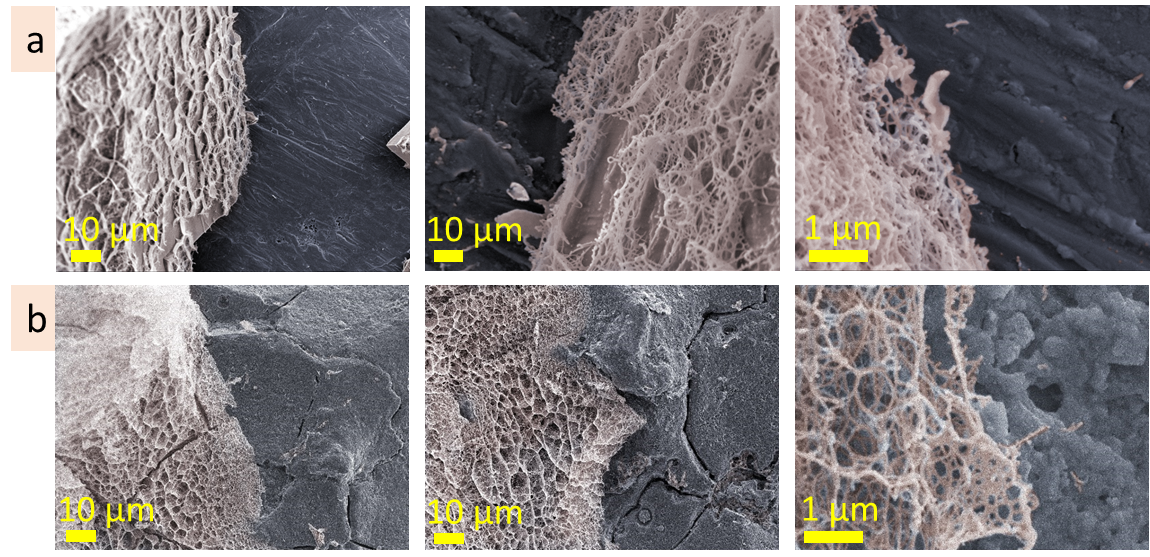


**Figure S19**. Cryo-SEM coloured images of agarose (0.3 wt%) microstructures after exposition on smooth (a) and porous (b) substrates. Blue colour corresponds to substrate, beige – to polysaccharide microstructures.


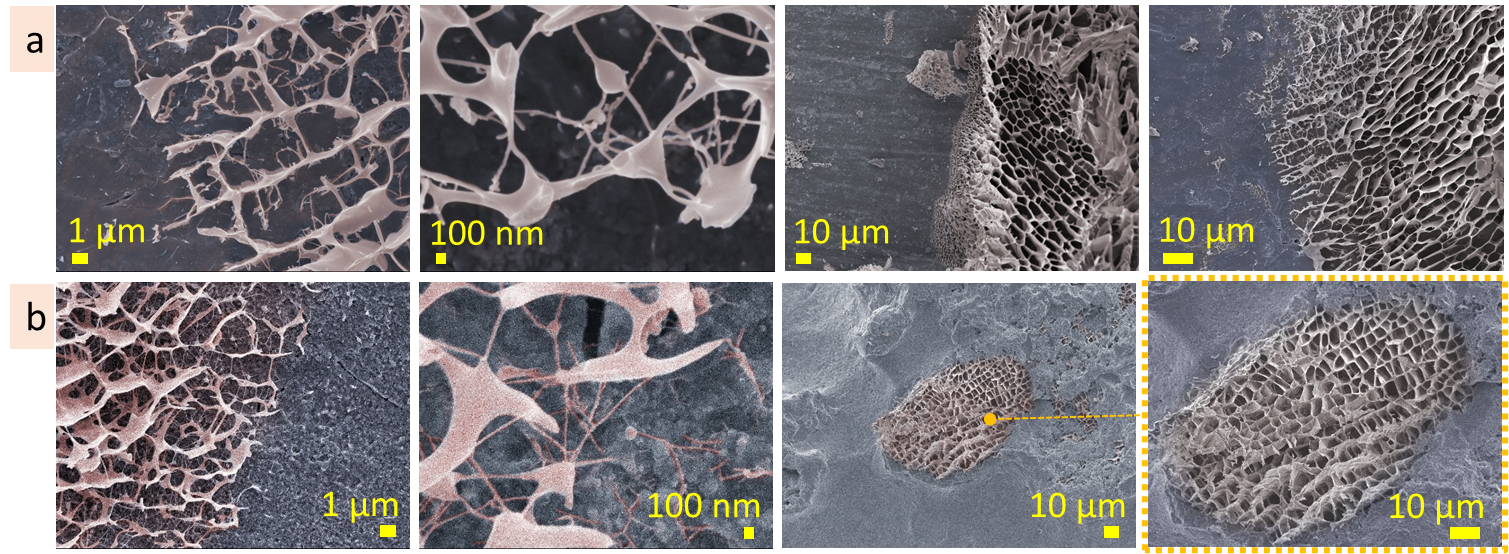


**Figure S20.** Cryo-SEM coloured images of alginic acid (5 wt%) microstructures after exposition on smooth (a) and porous (b) substrates. Blue colour corresponds to substrate, beige – to polysaccharide microstructures.


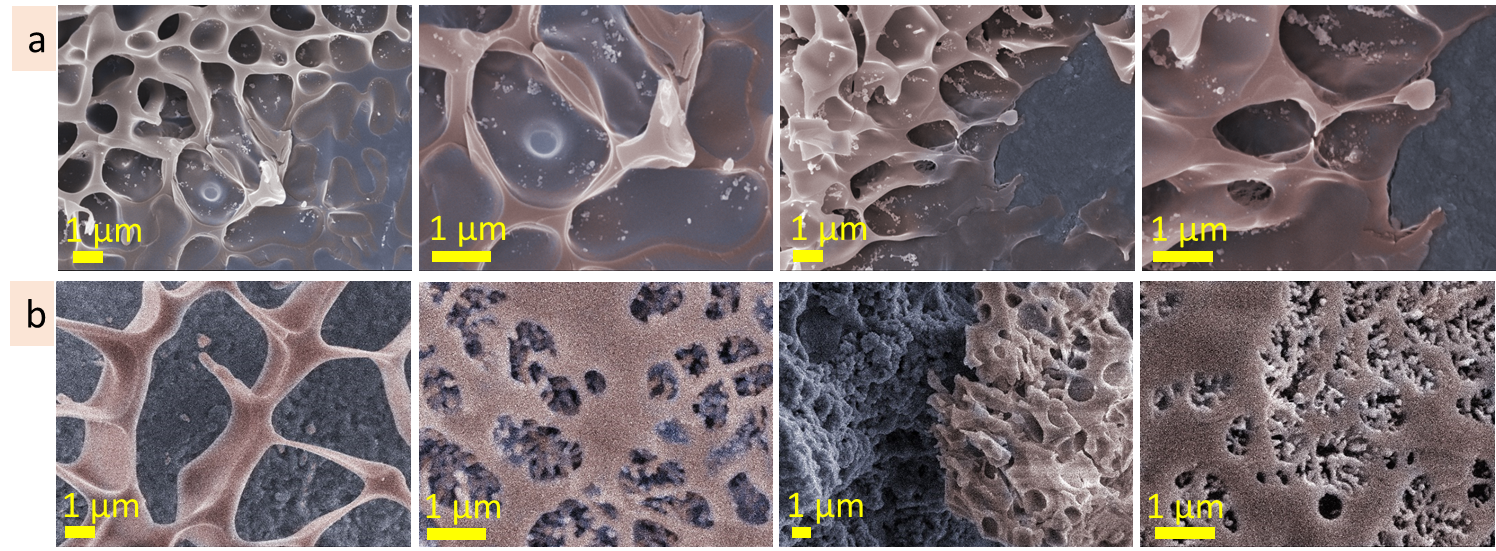


**Figure S21.** Cryo-SEM images of gum Arabic (20 wt%) microstructures after exposition on smooth (a) and porous (b) substrates. Blue colour corresponds to substrate, beige – to polysaccharide microstructures.


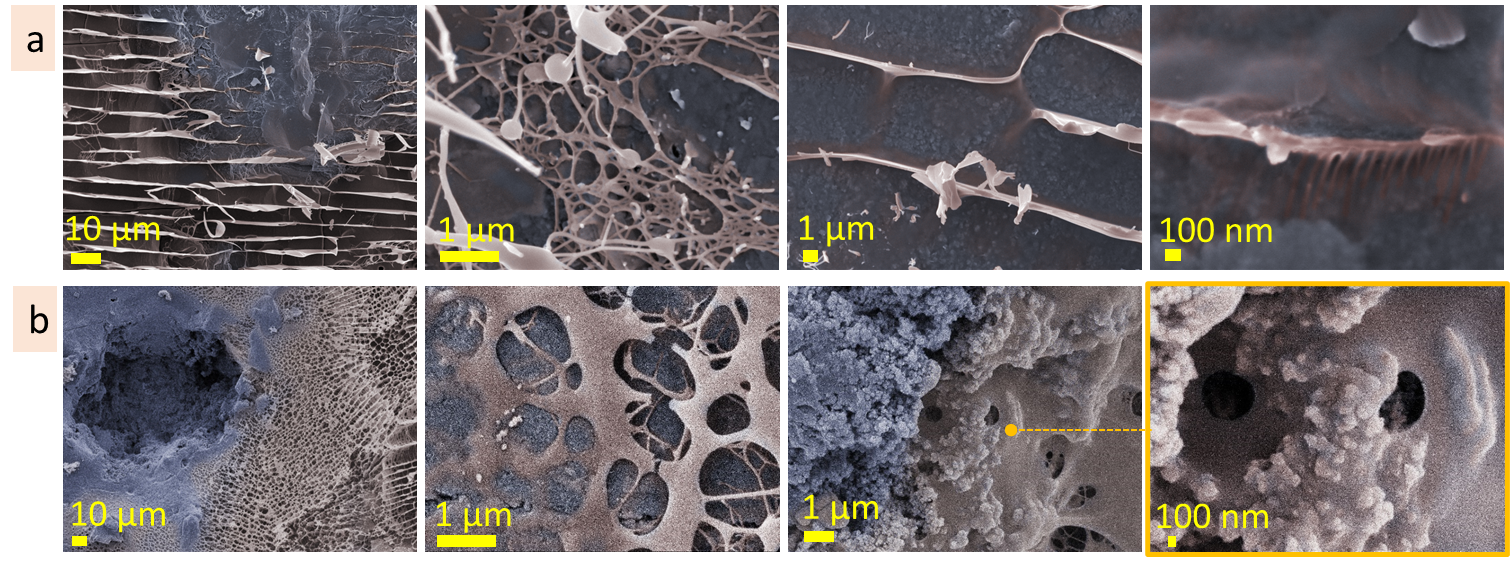


**Figure S22**. Reorganisational ability: cryo-SEM images of hyaluronic acid (1 wt%) microstructures after exposition on smooth (a) and porous (b) substrates. Blue colour corresponds to substrate, beige – to polysaccharide microstructures.


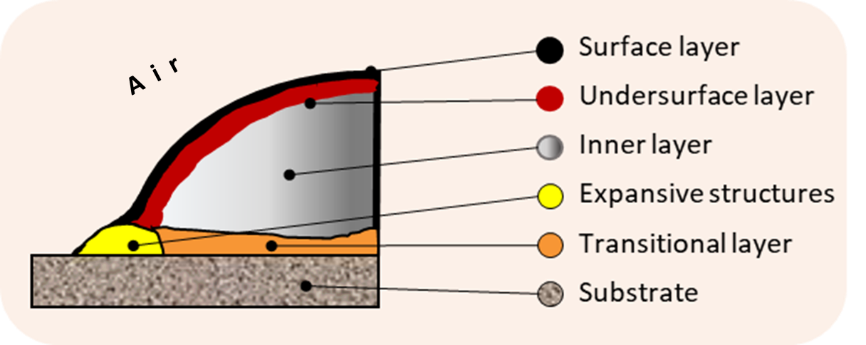


**Figure S23.** Scheme of the microstructure layers of polysaccharide solutions investigated by means of CLSM and cryo-SEM techniques.

**References**

1. Bhamidi, S. *et al.* The identification and location of succinyl residues and the characterization of the interior arabinan region allow for a model of the complete primary structure of Mycobacterium tuberculosis mycolyl arabinogalactan. *J. Biol. Chem.* **283**, 12992–13000 (2008).

2. Bhamidi, S., Scherman, M. S. & McNeil, M. R. Mycobacterial cell wall Aabinogalactan: a detailed perspective on structure, biosynthesis, functions and drug targeting. *Bact. Polysaccharides Curr. Innov. Futur. Trends* 39–65 (2009).
